# Short-term effect of a smartphone application on the mental health of university students: A pilot study using a user-centered design self-monitoring application for mental health

**DOI:** 10.1371/journal.pone.0239592

**Published:** 2020-09-25

**Authors:** Kosuke Kajitani, Ikumi Higashijima, Kosuke Kaneko, Tomoko Matsushita, Hideaki Fukumori, Daewoong Kim

**Affiliations:** 1 Center for Health Sciences and Counseling, Kyushu University, Fukuoka, Japan; 2 Content and Creative Design Course, Department of Design, Graduate School of Design, Kyushu University, Fukuoka, Japan; 3 Cybersecurity Center, Kyushu University, Fukuoka, Japan; 4 Department of Content and Creative Design, Faculty of Design, Kyushu University, Fukuoka, Japan; Chiba Daigaku, JAPAN

## Abstract

**Background:**

Despite the widespread recognition of the importance of mental health in young people, only a small proportion of young people with a mental disorder, including university students, receive mental health care.

**Objective:**

We developed a smartphone application (Mental App) for the university students and examined the effects of the app on their mental health.

**Methods:**

The app was designed according to a questionnaire survey conducted before this study. The Mental App was installed on the students’ smartphone and the psychological tests (the Link Stigma Scale, the Center for Epidemiologic Studies Depression Scale, and the 12-item General Health Questionnaire) were performed on the same day. After using the App for two weeks, the students completed a questionnaire survey and underwent the same psychological tests. We compared the results between the app user and non-user group.

**Results:**

A total of 68 students participated, of which 57 students completed the study (app user group, n = 28; control group, n = 29). The mean number of days spent using the app was 5.66 ± 3.16 (mean ± SD). The mean total screen time of the app was 9:03 ± 06:41(min:sec). The mean number of total actions (screen taps or swipes) was 161.91 ± 107.34. There were no significant between-group differences in the ΔLink Stigma Scale score (-0.11 ± 4.28 vs. -0.59 ± 3.30, *p* = 0.496) or the ΔCenter for Epidemiologic Studies Depression Scale score (-4.39 ± 7.13 vs. -2.07 ± 8.78, *p* = 0.143). There was a significant between-group difference in the ΔGeneral Health Questionnaire score (-2.21± 2.23 vs. -0.17 ± 2.69, *p* = 0.007).

**Conclusions:**

This non-randomized controlled pilot study indicates that the app we developed, may provide effective mental health care for university students, even in the short-term.

Trial registration: UMIN000040332

## Introduction

Mental health problems are a growing public health concern worldwide. Around 14% of the global disease burden is attributed to neuropsychiatric disorders, which primarily include depression, alcohol-substance abuse, and psychosis [1]. Although the burden of mental health disorders spans across all age groups, the highest proportion of disability-adjusted life years occurs in adolescents and young to middle-aged adults (aged 10–29 years) [2]. An estimated 10–20% of children and adolescents experience mental disorders worldwide [3]. In addition, half of all lifetime mental disorders start by the mid-teens and three quarters by the mid-20s [4, 5]. Suicide is also a serious public health problem that affects many young people and is the second leading cause of mortality in people aged 10 to 24 years [6]. Suicidal behavior is closely related to the presence of mental disorders. A meta-analysis of psychological autopsy studies using a profiling method that reconstructs a person’s psychological state before their death showed that 87.3% of suicide cases had been diagnosed with a mental disorder [7]. Given that mental disorders and suicide are major health problems for young people, enacting mental health measures for young people is an urgent issue.

Despite the widespread recognition of the importance of mental health promotion and prevention of psychiatric disorders in young people, the current evidence indicates that only a small proportion of young people with a mental disorder receive mental health care [8, 9]. Similarly, the World Health Organization reported that only 16.4% of the college students with mental disorders received a 12-month healthcare treatment for their mental disorders [10], which suggests that university students are perhaps hesitant to consult a psychiatrist or psychologist for their mental health problems. Furthermore, online surveys completed by college students at 26 campuses in the US showed that only half of the students with depression received adequate care [11]. There are several explanations as to why young people do not seek treatment for their mental health problems: 1) They do not pay attention to their mental health; 2) They are not aware of any consultation systems including medical services; 3) They are embarrassed to consult with others about their personal problems (fear of stigmatization); 4) They are too busy to consult with mental health specialists; and 5) They have restricted mobility due to physical conditions or apathy [12, 13]. One of the most cited barriers to treatment-seeking behavior is the stigma attached to mental health disorders and those who have received treatment for these issues. Furthermore, it has been suggested that the stigma attached to mental disorders is more pronounced in young people than the elderly [14]. Addressing these barriers to treatment could help university students consult with others about their mental health problems. Without treatment, students with mental health disorders are at a higher risk for lower grade point averages, school dropout, and unemployment than those without such disorders [15]. Thus, promoting early interventions for mental disorders could improve the quality of life of university students with mental health problems.

With the advances in information and communications technology, digital tools provide new avenues for changing health-related behaviors and managing chronic conditions [16]. In particular, smartphone applications (apps) are being widely used to support health care; it has been estimated that one-third of cellphone owners use mobile phones to obtain health information and 19% of smartphone users have at least one health app [17]. With the increasing use of mobile phones, mobile interventions have become a viable option to treat individuals with mental health problems. For young people, including university students, smartphones and apps are familiar tools that offer an effective and ubiquitous platform for delivering mental health information [18]. Many studies have examined the effect of smartphone apps for mental disorders, including depression, anxiety disorder, post-traumatic stress disorder (PTSD), addictive disorders (alcoholism and tobacco addiction), and developmental disorders [19–22]. There are now a variety of apps available for different purposes, including cognitive behavioral therapy (CBT), self-monitoring, screening of mental disorders, psychoeducation, and mindfulness [23–25]. Thus, smartphone apps have clinical relevance; however, currently, no mental health app with a user-centered design exists for university students.

To increase university students’ mental health support, we independently developed a smartphone app called the ‘Mental App’, which was designed for university students according to the results obtained from a questionnaire [26]. This user-centered designed app was equipped with a self-monitoring and self-screening function for mental disorders aimed to tackle the aforementioned obstacles (i.e., lack of attention to their mental condition, a paucity of information about consulting systems, lack of knowledge about mental illness, and not having enough time for consultation). In the present study, using psychological tests, we examined the app’s effect on the mental state of university students. Log data were also collected to assess the Mental App’s usage, including the frequency and behavioral patterns. Finally, we conducted a questionnaire survey on the overall design impression and usability of the Mental App.

## Materials and methods

### Study design

**Fig 1:**
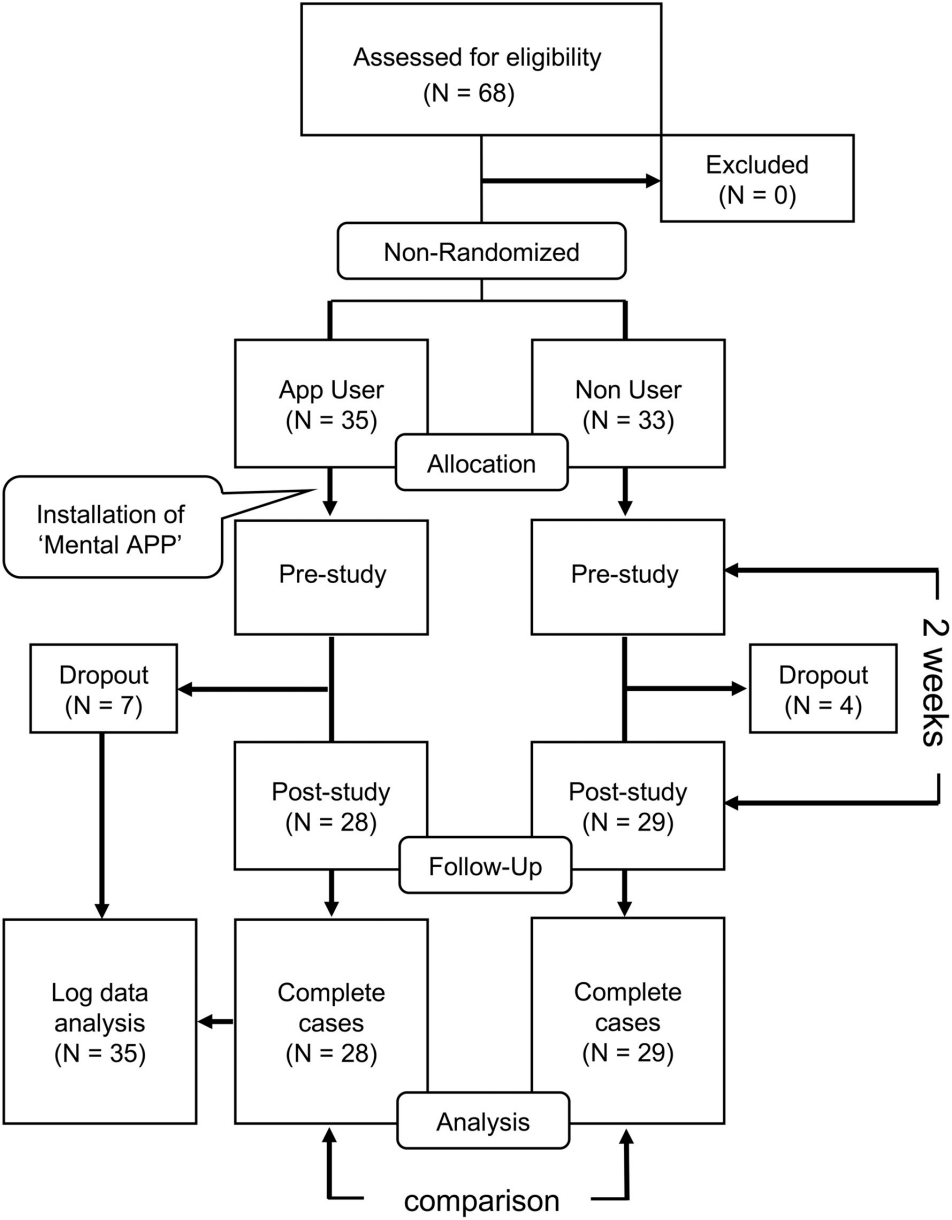
Flowchart of the recruitment process and the study design.

Recruitment for this study took place from December 21st, 2018, to September 6th, 2019. We did not register this study as a clinical trial before the enrolment of participants started, as we could not foresee any potential safety issues including any health problems. The Ethics Committee did not identify any safety risks and therefore did not require this study to be registered as a clinical trial. We retrospectively registered this study at the UMIN clinical trials registry (UMIN000040332), after being reminded that our study design falls under the WHO’s definition of a clinical trial. All ongoing and related trials for this intervention are hereby registered. This was a non-randomized controlled interventional study that evaluated the effect of our smartphone app on the mental health of university students. Participants were divided into an app user group and a non-user group. It was up to the subjects to decide whether they wanted to join the app user or the non-user group. The smartphone app (Mental App), which was developed independently by us, was installed on the students’ smartphones. The app was distributed using the ‘Apple Developer Enterprise Program’, which is a program for large organizations (e.g., companies, schools, and hospitals) for developing their internal apps and deploying them to their employees. For this reason, the installation of the app was possible only via the local area network of Kyushu University. Psychological tests (the Link Stigma Scale, LSS; the Center for Epidemiologic Studies Depression Scale, CES-D; and the 12-item General Health Questionnaire, GHQ-12) were conducted on the same day as the installation of the app (pre-study). After using the Mental App for two weeks, we conducted a questionnaire survey regarding the app’s overall design and usability. We also repeated the previously conducted psychological tests during this time (post-study). Fig 1 shows the study design of this project. For log data analysis, we collected the log data from all app users. To compare psychological test results, we used data from subjects who had completed the post-study.

### Participants

All participants were students who volunteered to participate and were recruited from the University’s Interdisciplinary Graduate School of Engineering Sciences and the Faculty of Arts and Science. In the psychology class, we explained the study’s outline and asked for volunteers. Written informed consent was obtained from each participant after providing them with information about the study. The inclusion criteria of this study were as follows: participants were 1) university students; 2) iPhone users; and 3) understood Japanese. The Ethics Committee of the Faculty of Arts and Science and Center for Health Sciences and Counseling at Kyushu University approved this study.

### Smartphone application ‘Mental APP’

The smartphone application, “Mental APP”, was used in this study. We independently developed the Mental APP for the university students according to the results of a questionnaire survey, completed prior to this study [26] which determined the colors, font sizes, button size, and icons preferred by the university students.

**Fig 2:**
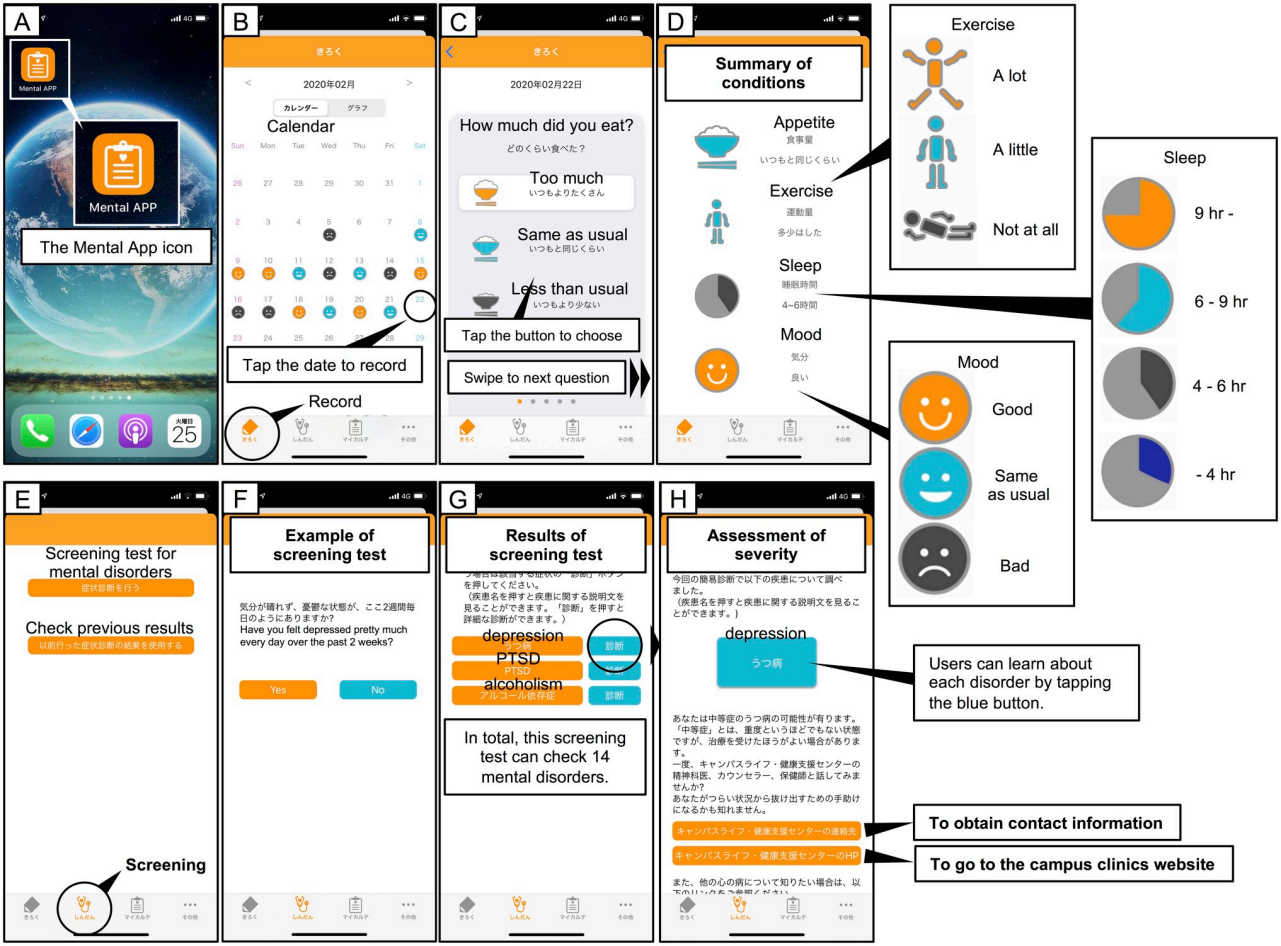
Screenshots of Mental App. Since the app is only available in Japanese, English captions are shown in the screenshots. (A) The Mental App icon. A magnified view is also shown. (B) The main screen of the record function. A calendar system was used to record daily conditions. Users can tap the date to record the conditions of that day. (C) Recording daily conditions. The screenshot shows a screen asking about users’ appetite for the day. Users choose the extent of their appetite by tapping the corresponding button. Users can swipe the screen to go to the next question. (D) The summary of the conditions of the day. In the record function, users can record their daily appetite, exercise, sleep time, and mood. (E) The main screen of the screening function for mental disorders. Users can start the screening test for mental disorders. (F) Example of the screening test. The screenshot shows the screening test for depression. (G) Results of the screening test. If the screening test was positive, more detailed tests for each disorder were offered to determine the severity. (H) Assessment of the severity of each mental disorder. According to the severity, the app offered the users advice and information (e.g., the contact information of the campus clinic or the nearest clinics, the website of campus clinic, a self-care website, and contact information of public consulting services). Furthermore, users could learn about each disorder by tapping the blue button.

The Mental App consisted of three major functions: 1) a self-monitoring function, 2) a self-screening function for mental disorders, and 3) a referral function for a psychiatrist or other consultation service. In the self-monitoring function, participants recorded their daily conditions, including appetite (too much, the same as usual, less than usual), sleep (more than 9 hours, 6–9 hours, 4–6 hours, less than 4 hours), exercise (a lot, a little, not at all), and mood (good, the same as usual, bad). After completing a minimum of one week, the app provided advice to users on how to improve their physical and mental conditions according to the results of the daily record (e.g. “You should eat more”, “You had enough time to sleep”, and “You need to exercise more”). In the self-screening function for mental disorders, participants could conduct a brief screening for the 14 mental disorders (depression, suicidal ideation, bipolar disorder, psychotic state, generalized anxiety disorder, panic disorder, agoraphobia, social anxiety disorder, obsessive-compulsive disorder, PTSD, drug addiction, alcoholism, anorexia nervosa, and bulimia nervosa). The self-screening for mental disorders was based on the multi-item measures of each mental disorder. According to the severity of each mental disorder, the app offered advice to users in the referral function (e.g., provide contact information of the campus clinic or the nearest clinics, the website of campus clinic, the self-care website, and/or contact information of public consulting services). Users could search the location and contact information provided by “Google Maps”, linked within the Mental App. Furthermore, in this referral function, participants could learn more about each of the mental disorders (e.g., the definition of the disorder, causes, symptoms, and treatment). The app design process is shown in Fig 2.

To design the aesthetic elements for the app, Sketch version 41.2 (Bohemian Coding, London, UK) was used. The app was written using Xcode version 8.2.1 and 10.1 (Apple Inc.) and the development language was swift 3.0 (Apple Inc.). The target phone models for the app were iPhone 4s, 5, 5s, 5c, 6, 6 plus, 6s, 6s plus, SE, 7, 7 plus, 8 plus, X, XS, XS Max, and XR (Apple Inc.). Mental App was only available in Japanese.

To check app usage, we collected the log data on when (the date and time) the app was used and what button the users tapped or swiped while using the app. Based on these log data, we calculated the number of days the app had been used, the screen time, and the number of actions (taps and swipes). The program for log data collection was created by the source code editor Visual Studio Code version 1.24.1 (Microsoft Corporation, Redmond, WA, USA) using the programming language PHP version 7.2.14.

### Psychological tests

#### Link Stigma Scale

The LSS was originally developed by Link and colleagues to measure the public stigma [27, 28]. We used the Japanese version of the LSS [29], which comprises 12 items and measures the perceived community residents’ attitudes towards people with mental illness. For example, “Most people believe that a person who has been in a mental hospital is just as intelligent as the average person”. Agreement with items was scored on a 4-point Likert scale (strongly disagree, disagree, agree, and strongly agree). We constructed an index of perceived stigma by coding each response as 1, 2, 3, or 4 (with higher numbers corresponding to higher perceived stigma).

#### The Center for Epidemiologic Studies Depression Scale

The CES-D is a commonly used self-report measure of depressive symptoms in the general population [30]. The CES-D is a 20-item measure that asks subjects to rate how often, over the past week, have they experienced symptoms associated with depression. Items are scored on a 4-point Likert scale (0 = rarely or none of the time, 1 = some or little of the time, 2 = moderately or much of the time, 3 = most or almost all the time), and thus, the total CES-D score ranges from 0 to 60, with higher scores indicating more severe depressive symptoms.

#### The 12-item General Health Questionnaire

The GHQ, developed by Goldberg, is one of the most widely used screening instruments for mental health [31]. The original version of the GHQ consists of 60 items, and short versions of the GHQ include the GHQ-12, GHQ-20, GHQ-28, and GHQ-30. In the present study, we used the Japanese version of the GHQ-12 since it is simple but reliable enough to evaluate psychological distress and social dysfunction [32]. Each of the 12 items of the GHQ-12 asks respondents if they recently had experienced a particular symptom or behavior. Each item is rated on a four-point scale (less than usual, no more than usual, a little more than usual, or much more than usual). In the present study, we employed a bi-modal method of scoring (0-0-1-1), and thus, the total GHQ-12 score ranged from 0 to 12, with higher scores indicating worse mental health.

### Questionnaires

After using the app, we conducted a questionnaire survey to measure the participants’ impressions of the app. This included questions about the button size, font size, color arrangements, design of the app, the operability of the app, and future usage for this app.

### Statistical analysis

Data were analyzed using IBM SPSS Statistics version 24.0 (IBM Corporation, Armonk, NY, USA). Chi-square or Fisher’s exact tests were used to compare the categorical variables between the groups and independent samples *t*-tests were used to compare the continuous variables (Table 1). Between-group comparisons of psychological test scores were performed using the Mann-Whitney *U* test (Table 3 and Fig 4). Wilcoxon single-rank tests were used to compare the pre-study and post-study results (Table 3). We conducted the univariate ANCOVAs to test for differences between the groups (app user and non-user), with pre-test values as the covariate. Statistical significance was set at *p* < .05.

**Table 1:** Demographics of subjects who completed the post-study.

	app user group	non-user group	*p*-values
Subjects (n)	28	29	—
Age (year) *	21.8 ± 1.60	21.7 ± 3.30	0.851
Sex (male/female) ^#^	20/8	20/9	0.839
Academic Course ^$^			0.982
Undergraduate	17	17	—
Master’s	11	11	—
Doctorate	0	1	—
Major ^$^			0.364
Humanities/liberal arts	9	7	—
Science/engineering	18	17	—
Medicine/pharmacology	1	4	—
Other	0	1	—

*Independent samples *t*-test;

^#^Chi-square test;

^$^Fisher’s exact tests.

## Results

In total, 68 students participated in this study (app user group, n = 35; non-user group, n = 33). Eleven students did not participate in the post-study (app user group, n = 7; non-user group, n = 4). Therefore, we used data from the 57 subjects (app user group, n = 28; non-user group, n = 29; Fig 1). The demographic characteristics of the study participants who completed this study are shown in Table 1. There were no significant between-group differences in age, sex, academic degree, and major in the university.

**Fig 3:**
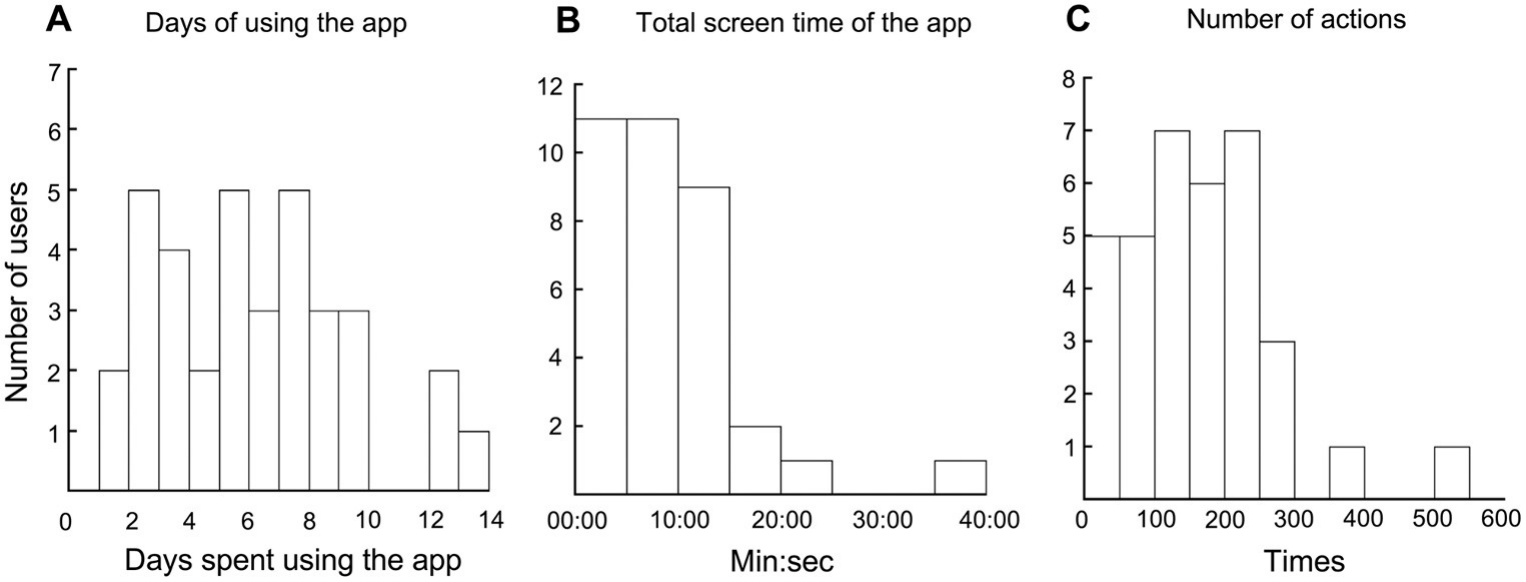
Histograms of log data. (A) Histogram of days spent using the app. (B) Histogram of screen time of the app. (C) Histogram of the total number of actions (screen taps or swipes).

First, we analyzed the log data to check how often the subjects used the Mental App (Table 2). For this log data analysis, we examined data from 35 app users, which included subjects who did not complete the post-study. The mean number of days spent using the app was 5.66 ± 3.16 (mean ± SD; Fig 3A). Forty percent of the subjects used the app for more than 7 days. The mean total screen time of the app was 9:03 ± 6:41 (min:sec). A histogram of the app’s screen time is shown in Fig 3B. Eighty-nine percent of subjects used the app for less than 15 min. The mean number of total actions was 161.91 ± 107.34. Sixty-six percent of subjects tapped or swiped the screen 100 to 300 times (Fig 3C). All subjects used the self-monitoring function, and 23 out of the 35 subjects used the self-screening function (Table 2).

**Table 2:** Summary of log data.

Log data	N = 35
Days spent using the app (days)	5.66 ± 3.16*
Total screen time of the app (min:sec)	9:03 ± 6:41*
Number of actions (times)	161.91 ± 107.34*
Use of the ‘self-monitoring function’ (yes/no)	35/0
Use of the ‘self-screening function’ (yes/no)	23/12

*mean ± SD.

Table 3 shows the mean scores of all psychological tests. There were no significant between-group differences at either pre- or post-study time points (pre-study LSS score: app user vs. non-user, 28.9 ± 7.51 vs. 29.6 ± 5.19, *p* = 0.548; pre-study CES-D score: app user vs. non-user, 22.0 ± 12.8 vs. 16.2 ± 12.1, *p* = 0.070; pre-study GHQ-12 score: app user vs. non-user, 4.86 ± 3.54 vs. 3.10 ± 2.85, *p* = 0.056; post-study LSS score: app user vs. non-user, 28.8 ± 6.38 vs. 28.9 ± 5.98, *p* = 0.823; post-study CES-D score: app user vs. non-user, 17.6 ± 11.8 vs. 15.8 ± 11.2, *p* = 0.330; post-study GHQ-12 score: app user vs. non-user, 2.64 ± 3.00 vs. 2.93 ± 3.37, *p* = 0.826).

**Table 3:** Results of the psychological tests.

	Pre-study		Post-study	
	app user group	non-user group	app user group	non-user group
LSS score	28.9 ± 7.51	29.6 ± 5.19	28.8 ± 6.38	28.9 ± 5.98
CES-D score	22.0 ± 12.8	16.2 ± 12.1	17.6 ± 11.8*	15.8 ± 11.2
GHQ-12 score	4.86 ± 3.54	3.10± 2.85	2.64 ± 3.00*	2.93 ± 3.37

Mann-Whitney *U* tests were used for between-group (app user group vs. non-user group) comparisons; Wilcoxon single-rank tests were used for within-group (pre- vs. post-study) comparisons; CES-D score: pre-study vs. post-post in the app user group, *p < .01; GHQ-12 score: pre-study vs. post-study in the app user group, *p < .01.

We also compared the test scores between the pre-study and post-study time points. There was a significant within-group difference in the CES-D score for the app user group (22.0 ± 12.8 vs. 17.6 ± 11.8, *p* = 0.004). There was also a significant within-group difference in the GHQ-12 score in the app user group, whereby the pre-study score was significantly higher than that in the post-study score (4.86 ± 3.54 vs. 2.64 ± 3.00, *p* < .001). There was no significant within-group difference in the LSS score in the app user group (28.9 ± 7.51 vs. 28.8 ± 6.38, *p* = 0.556). In the non-user group, there were no significant within-group differences for any of the test scores (non-user LSS score: 29.6 ± 5.19 vs. 28.9 ± 5.98, *p* = 0.365; non-user CES-D score: 16.2 ± 12.1 vs. 15.8 ± 11.2, *p* = 0.161; non-user GHQ-12 score: 3.10± 2.85 vs. 2.93 ± 3.37, *p* = 0.472).

**Fig 4:**
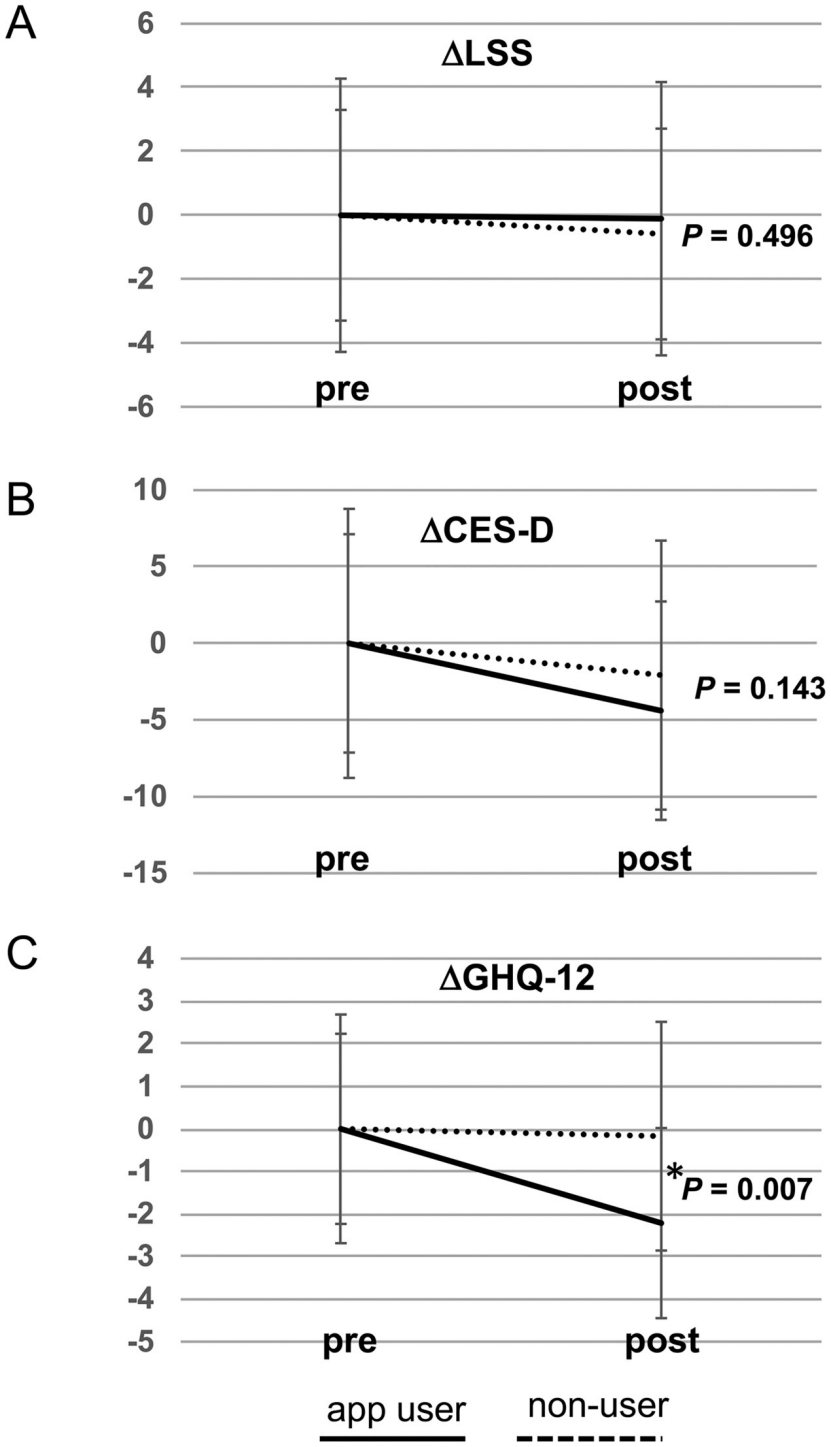
Comparison of the difference in the psychological test scores before and after the intervention (Δvalue) between app users and non-users. (A) There was no significant difference in the Δvalue of the LSS score (ΔLSS) between the app user and non-user groups (*p* = 0.496). (B) There was no significant between-group difference in the Δvalue of the CES-D score (ΔCES-D; *p* = 0.143). (C) Comparison of the Δvalue of the GHQ-12 (ΔGHQ-12) between the app user and non-user groups. The app user group showed a significantly greater difference score than that of the non-user group (*p* = 0.007). Mann-Whitney *U* test, *p < .05.

Next, we compared the variation before and after the intervention (Δvalue) in each psychological test between the app user and non-user group to examine the effect of the Mental App on each psychological test. There was no significant between-group difference in the ΔLSS (-0.11 ± 4.28 vs. -0.59 ± 3.30, *p* = 0.496; Fig 4A). Similarly, there was no significant between-group difference in the ΔCES-D (-4.39 ± 7.13 vs. -2.07 ± 8.78, *p* = 0.143; Fig 4B). However, there was significant between-group difference in the ΔGHQ (-2.21± 2.23 vs. -0.17 ± 2.69, *p* = 0.007; Fig 4C).

To exclude the possibility that the difference in the ΔGHQ was caused by a regression to the mean, univariate ANCOVA was used to assess the effect of using the Mental App. The ANCOVA analysis found a statistically significant difference [F (1, 54) = 5.72, *p* = 0.020] between the app user and non-user group in GHQ-12. However, there were no significant differences between the app user and non-user group in the LSS [F (1, 54) = 0.11, *p* = 0.74] and CES-D [F (1, 54) = 0.12, *p* = 0.74] (Table 4).

**Table 4:** Comparison of app user and non-user group using ANCOVAs in post-test with pre-test as covariates.

measures	groups	M_adj_ (± SD)	*F*	*p*	η^2^	95% CI	
						lower	upper
LSS	app user	29.1 ± 6.68	0.11	0.74	0.02	27.8	30.4
	non-user	28.8 ± 6.56				27.5	30.1
CES-D	app user	15.2 ± 1.39	0.12	0.74	0.02	12.5	18.0
	non-user	15.9 ± 1.36				13.2	18.6
GHQ-12	app user	2.02 ± 0.44	5.72	0.02*	0.10	1.13	2.91
	non-user	3.53 ± 0.44				2.66	4.40

M_adj_: Adjusted means of post-study, 95% IC: 95% confidence interval, *p*: *p*—value, * < .05.

Finally, we surveyed the users’ impressions of the Mental App using a questionnaire. Most users gave the app a good evaluation (Table 5). For example, 92.8% of users were “slightly satisfied” or “satisfied” with the overall design of the app and 85.7% were “slightly satisfied” or “satisfied” with the app’s operability. Furthermore, 60.7% of the users reported that they would like to continue using the app.

**Table 5:** Participants’ impressions of the Mental App.

Button size	Too small	Slightly small	Right size	Slightly large	Too large
	0	2	23	1	2
Size of font size	Too small	Slightly small	Right size	Slightly large	Too large
	0	3	24	0	1
Color arrangement	Dissatisfied	Slightly dissatisfied	Neither	Slightly satisfied	Satisfied
	0	0	1	7	20
Overall app design	Dissatisfied	Slightly dissatisfied	Neither	Slightly satisfied	Satisfied
	0	0	2	7	19
App operability	Dissatisfied	Slightly dissatisfied	Neither	Slightly satisfied	Satisfied
	1	2	1	8	16
Do you want to continue using this app?	No	Neither	Yes		
	3	8	17		

## Discussion

In the present study, we independently developed a smartphone app to provide and assess the mental health support of university students. This user-centered designed app was equipped with a self-monitoring function (daily user’s condition: appetite, exercise, sleep, and mood), self-screening function (14 mental disorders), and a referral function that referred the users to psychiatrists or other consultation services. Furthermore, we examined the effect of the app on the mental health of students. The app usage improved the GHQ-12 score, although the subjects used the app for only 2 weeks. There were no improvements observed for the depressive symptoms (CES-D score) or the prejudice towards people with mental illness (LSS score). This indicates that the current app may provide effective mental health care, even in the short-term.

Some observational studies have examined the mental conditions of university students using a smartphone app; however, this is the first interventional study that independently developed an app for university students and evaluated its effect on their mental health. Sano et al. developed a new system that provided objective physiological and behavioral assessments using smartphones and wearable sensors [33]. In that study, the authors recruited 201 college students and collected data from wearable sensors (skin conductance, skin temperature, three-axis acceleration, activity levels, and sleep/wake patterns), a smartphone app (location, receivers, senders, and timings of calls and SMS text messages, the screen’s on or off durations, and phone app usage), and electronic diaries with two entries per day. Furthermore, each student filled out self-reported questionnaires on stress and mental health before and after the study. The authors found that the data obtained from each device could identify stress levels and mental conditions of the college students with high accuracy; however, the study was observational and did not compare the efficacy of an app on mental health with a control group. Geyer et al. also developed an app for college students to examine how emotional ratings during social interactions later predicted the perceptions of those interactions from the viewpoint of social anxiety [34]; however, this was also an observational study. Using commercially available apps, Huberty et al. examined the efficacy of the mindfulness meditation app “Calm” on mental health in college students [35]. In this randomized controlled trial (RCT), the use of the app resulted in significant improvements in all outcomes, including perceived stress levels, mindfulness scores, and self-compassion scores.

Apart from research conducted in university students, many other interventional studies have reported improvements in psychiatric symptoms after using smartphone apps in other populations. For example, Mantani et al. evaluated the efficacy of a CBT app as adjunctive therapy in patients with antidepressant-resistant major depression. In that study, a medication switch plus CBT app group showed significant improvements in depression scores compared to a control group (medication switch alone) [36]. Arena et al. reported that a cognitive training app and problem-solving therapy app group exhibited greater benefits on mood than the control group [37]. Ivanova et al. investigated the efficacy of an Internet-delivered acceptance and commitment therapy-based treatment program using a smartphone on anxiety disorders; the treatment group had reduced general and social anxiety disorder, but no decrease in panic symptoms compared to the waiting-list group [38]. Overall, several interventional studies have examined the effect of CBT (in a broad sense) using smartphone apps and confirmed its efficacy [23, 39].

The main function of our app was the self-monitoring system (daily appetite, exercise, sleep, and mood), and our app did not include a CBT program. According to previous reports, it is still controversial as to whether self-monitoring by a mobile device is effective for reducing psychiatric symptoms. For example, Reid et al. conducted an RCT that examined the mental health benefits of self-monitoring using a mobile phone. The authors found no significant differences in depression, anxiety, or stress scores between an intervention and control group, although the use of the mobile phone program was significantly associated with an increase in the emotional self-awareness (ESA) score [40]. Faurholt-Jepsen et al. examined whether the use of daily electronic self-monitoring using smartphones could reduce depressive and manic symptoms in patients with bipolar disorder; however, there were no significant effects of self-monitoring on depressive or manic symptoms [41]. In contrast, an RCT conducted by Kauer et al. reported that self-monitoring mood, stress, and coping strategies by mobile phone increased ESA and that this ESA increase was associated with a significant decrease in depressive symptoms [42]. Bakker et al. examined the efficacy of a self-monitoring mobile phone app that provided daily prompts to complete a short mood questionnaire and found that app engagement ratings predicted decreases in depression and anxiety. In addition, the authors found that the app increased the mental well-being scale score and that these effects were mediated by increases in ESA [43]. In the present study, the CES-D scores decreased after using the app, although this decrease (Δvalue) was not significant compared to that in the non-user group. Furthermore, we found that the GHQ-12 score, which is a measure of current mental health, improved significantly in the app user group compared to that in the non-user group. Considering previous results from studies that have used a self-monitoring app, it is possible that our app improved the GHQ-12 score via an increased ESA. ESA is the ability to understand one’s own emotions, which can improve emotional self-regulation [44]. A low ESA is common in both anxiety and depressive disorders [45]. In addition, given that a high GHQ-12 score is associated with psychiatric symptoms, including depression and anxiety, our findings of a decreased GHQ-12 score in the app users may reflect an increased ESA. Moreover, our app provided contact details for psychiatrists and other consultation services when necessary and this referral function may have also relieved the users’ anxiety. However, there is not enough data to clarify by what mechanism our app improved the mental health of its users and this warrants further study.

In general, the cut-off point for CES-D is 15/16 and that for GHQ-12 is 2/3; however, the mean scores of these tests were relatively high in our studies. There may be two reasons for this discrepancy. First, all subjects in the present study were student volunteers and some students with mental health problems may have participated in the study. Students with mental health problems may have been interested in our research due to concerns regarding their psychological state. Therefore, students with mental health problems may have raised the mean score of these psychological tests. Second, cultural background in Japan may have also further impacted the results of this study. Several studies report a relatively high score of CES-D and GHQ-12 in Japan. For example, Doi et al. performed GHQ-12 on the Japanese general adult population (1,808 subjects) and found that young adults (20–29 years) exhibited relatively high scores compared to the elders (men: 2.77± 2.86, women: 3.88± 3.16) [32]. Ohtsu et al. evaluated the mental health status of 1,619 medical students and found the mean (± SD) score of male students to be 3.00 (± 2.94) and that of female students to be 3.82 (± 3.10) [46]. Concerning CES-D, a previous study examined the prevalence of depression among 2,200 Japanese people using CES-D and found the mean (± SD) CES-D score to be 16.09 (± 8.61) [47]. Furthermore, another study in Japan showed that 60.1% of college students exceeded the cut-off point of CES-D (≥ 16) [48]. Therefore, the psychological tests that examine mental problems may tend to score relatively higher in Japan.

We also examined whether the app could improve the students’ stigma of psychiatric disorders. The app had a self-screening function for mental disorders that provided general information about each mental disorder. In the general information, the app explained that mental disorders are common and that anyone can develop a mental disorder; however, there were no differences in the LSS score between the app user and the control group. One RCT with 262 subjects found that E-learning improved the stigma against mental illness [49]. In that study, participants were sent weekly E-mails via personal computers for a month to help them complete an E-learning CD. In contrast, our app did not prompt students to use the self-screening function and only 23 out of 35 students spontaneously used this function. In addition, the duration of our intervention was shorter than this beforementioned study. Thus, it is possible that with a longer intervention time, we would have observed a larger anti-stigma effect. Given the scarcity of studies that have investigated the removal of stigma using digital devices, further research is required to confirm whether smartphone apps are a useful tool in this context.

Regarding adherence with smartphone apps for mental health, previous reports have been inconsistent. For example, Huberty et al. examined the effect of an 8-week mindfulness course in college students using a mobile app and only 78.6% (44/56) of the students completed the post-intervention survey [35]. Meanwhile, Mak et al. found that only 23.5% (508/2161) of the participants completed the assessment after the 4-week program [50]. Concerning the number of days spent using apps, Kenny et al. reported that on average, participants used the app on 4 out of 7 days in their study; they examined the efficacy of an emotional self-monitoring app [51]. Matthews et al. found that users of a mobile mood diary demonstrated significantly higher levels of compliance (mean 8.12 days) compared to a control group who charted their mood using a pen and paper (mean 5.44 days) [52]. In our study, 28 out of 35 subjects in the app user group (80.0%) completed the study and the average days spent using the app was 5.66 ± 3.16 days out of the 14 days of intervention. Considering that we did not prompt students to use the Mental App, adherence to our app is not necessarily poor compared to other apps used for mental health. That said, the conditions of the present study were different from those of previous reports. We developed the Mental APP based on a questionnaire survey conducted before this study and that may have resulted in good adherence as the app’s design was heavily influenced by the user’s input.

The present study has some limitations. First, this study was not an RCT, so the results may have been affected by the potential confounding factors. In our study, only iPhone users could participate in the intervention group (app user group) and other types of mobile phone users were allocated to the control group (non-user group). A previous study found that there may be minor differences in personality between iPhone and Android users and that such minor differences may reflect the differences in sociodemographic factors [53]. It is, therefore, possible that there were sociodemographic differences between the two groups that may have affected the present results. In the future, we would like to create an Android version of the Mental App to confirm the effect of the app on mental health in students and distribute this app to the university students of various backgrounds. Second, all participants were recruited from a single university. In addition, the sample size of this pilot study was relatively small and our findings may not be generalizable to the majority of the university students. Third, the duration of this study was too short to evaluate the efficacy of the Mental App. A longer observation period may reveal additional benefits or even adverse effects of our app. In particular, we think that a longer intervention time may be required for university students to experience changes in the stigma. Finally, the Mental App did not include any function that dealt with academic problems (e.g., academic performance, mild developmental disorders, harassment, and employment after graduating the university). In addition, students of various ages, including middle-aged graduate students, also attend the university. Therefore, it is necessary to further expand the app’s functions to encompass such additional problems and diverse users at the university.

## Conclusion

We independently developed a smartphone app for university students and the use of the app improved the GHQ-12 score. However, app use did not improve the CES-D or LSS scores. This pilot study indicates that a self-monitoring app could improve mental health in university students. Further studies are needed to evaluate the efficacy of our app, particularly rigorously controlled research designs such as RCTs, that have longer intervention periods. Given the mental health burden in young adults, smartphone apps could be a promising option for improving the mental health of university students.

## Supporting information

S1 Checklist TREND statement checklist. (PDF)

S1 Dataset (XLSX)

S1 File (DOCX)

S2 File (DOCX)
