# Supplementary material for: Short-term effect of a smartphone application on the mental health of university students: A pilot study using a user-centered design self-monitoring application for mental health
Source: PLoS One. 2020 Sep 25;15(9):e0239592. doi: 10.1371/journal.pone.0239592 (PMC7518576; doi:10.1371/journal.pone.0239592)
Supplement: S1 File — (DOCX) [file pone.0239592.s003.docx]

**研究計画書**

申請日:2015年12月24日

承認日:2016年2月9日

再申請日:2020年1月19日

再承認日:2020年2月21日

九州大学

キャンパスライフ・健康支援センター

准教授　梶谷 康介

**研究課題名**

スマートフォンアプリによる学生のメンタルヘルスケア向上に関する実証研究

**研究実施体制**

代表研究者 九州大学キャンパスライフ・健康支援センター ・准教授・梶谷康介

(代表研究者連絡先) 〒819-0395　福岡市西区元岡744

センター5号館5613室
         Tel: (個)092-802-5116
                 (代)092-802-5124

E-mail: [kkajitani@chc.kyushu-u.ac.jp](mailto:kkajitani@chc.kyushu-u.ac.jp)

分担研究者 九州大学芸術工学研究院・教授・金 大雄

九州大学サイバーセキュリティーセンター・准教授・金子 晃介

九州大学キャンパスライフ健康支援センター・准教授・福盛英明

九州大学キャンパスライフ健康支援センター・准教授・松下智子

九州大学キャンパスライフ健康支援センター・准教授・土本利架子

九州大学キャンパスライフ健康支援センター・講師・舩津文香

**研究背景**

大学生時代は青年期の後期にあたり、身体的には成熟する一方、Eriksonらが指摘するような「アイデンティティ確立」など精神的未熟さを残す時期でもある。つまり精神の成長が身体の成長から取り残され、心と体の成熟度に大きなギャップが生じるアンバランスな時期と言える。この心身のアンバランスさを反映してか、厚生労働省のデータによると青年期にあたる10代後半から20代前半における死因の第一位は自殺となっており、また自殺者の多くがうつ病や薬物依存などの精神疾患に罹患していたと考えられている(Arsenault-Lapierre, BMC Psychiatry, 2004)。申請者は大学の保健管理センターにおいてメンタルヘルスに問題を抱えている学生の相談・治療に従事している。当大学における過去10年間の学生の保健管理センター利用者数と自殺率を比較すると、驚くべきことにメンタルヘルスに関する学生の相談件数自体は、この10年横ばい又は低下傾向を示す一方、自殺率に関しては右肩上がりである(未発表データ)。自殺者の多くが精神疾患を発病しているという先行研究と考え合わせると、この結果は「メンタルヘルスに深刻な問題を抱えている多くの学生が、大学の保健管理センターを利用していない」という実態を浮き彫りにしている。申請者はこの事態を改善すべく、①学生がメンタルヘルスに関して興味を持つ、②自分のメンタルヘルスについて客観的に見つめる、③自らメンタルヘルス向上のためにセルフケアができるツールの開発が肝要と考え、本研究を考案した。

**研究目的・意義**

　本研究の目的は、学生のメンタルヘルスケアに有用なスマートフォン用アプリケーションを開発し、大学生のメンタルヘルスを向上させることにある。具体的には1.メンタルヘルスのセルフチェック機能を持つアプリの開発、2.開発したアプリを実際に大学生に利用していただき、メンタルヘルスケアに有用か否かを検討する、である。本研究が成功した場合の成果として、1.学生の保健管理センターなどの医療機関への受診率が上がる、2.その結果、精神疾患の早期発見や自殺防止効果が期待でき、3.また社会的な精神疾患に対するスティグマが軽減される、などが挙げられる。上記効果は個人だけでなく、コミュニティ全体への大きな貢献になると考えられる。また、本研究は対象をあくまで大学生としているが、このアプリケーションがメンタルヘルスケアに有効であることが実証できれば、他のコミュニティへの適応、例えば職場ストレスの評価と対応、高齢者やその介護者のメンタルヘルスモニタリングなどに様々な場面での応用が期待できる。

**研究方法と対象**

研究デザイン**:** 介入研究である。

資料・データ: 新たにデータを入手する。尚、他施設からのデータ提供は受けない。

対象者およびリクルート方法: 九州大学に所属する学生。スマートフォンアプリとメンタルヘルスに関する先行研究を参考に、対象者数は100名程度を想定している(Ludtke, Psychiatry Res, 2018)。リクルート方法としては、当センターを利用した学生への声かけや、授業で告知して参加を促す。

研究方法: (開発からブラッシュアップ) 研究初年度(2016年度)においては、まずアプリケーション(アプリ)の内容と構成を決める。アプリの構成は大きく分けて、1.プラットホームアプリ(本アプリの基盤となるアプリ)、2.初期アプリ(プラットホームアプリをインストールした時に付属するアプリ)、3.追加アプリ(ユーザーが必要に応じて追加できるアプリ)、に分類される。アプリケーション使用の主な流れは、最初に初期アプリに属する「簡易診断用アプリ」を起動させ、ユーザーが精神疾患に該当するか否かを評価する。簡易診断については精神疾患簡易構造化面接法(M.I.N.I.: Mini-International Neuropsychiatric Interview)を参考に作成する。この簡易診断用アプリで該当する疾患に関して、その疾患の重症度を評価する追加アプリのインストールを推奨する。例えば、うつ病の疑いがあればベックのうつ病尺度(ISBN: 0-8122-1032-8)をベースにした尺度をインストールさせる。該当する追加アプリでユーザーが罹患している疾患の重症度を評価し、その重症度に応じて、対処方法を教示する。例えば、ごく軽度の障害であれば、セルフケアや学内カウンセリングの紹介、中等度であれば学内保健管理センターの紹介、重度かつ緊急性が高い場合は周辺の医療機関を紹介するなど、それぞれの対処方法をユーザーに提示する。さらに必要に応じて、心の電話の電話番号、既存のマップアプリとの連動で近くの精神科クリニックまでのアクセス方法の表示など、相談機能を充実させる。尚、予算の制限のためコンテンツの一部を変更する可能性はある。

　2017年度は、研究の目標である大学生のメンタルヘルスを向上させるために、大学生の目線でコンテンツの制作とシステムの開発を次のプロセスで行う。

1.大学生が主に利用しているアプリと利用実態を調査分析し、本研究に適したインターフェースを開発する。

2.2016年度の詳細設計を反映したスマートフォン用プロトタイプコンテンツを制作する。

3.プロトタイプコンテンツを用いてユーザー評価を行う。評価の際にはユーザーがどのページにどれだけ滞在していたのかを記録したログデータを収集し、分析する。またアンケート調査による主観評価も行う。プロトタイプコンテンツのユーザーのリクルートは、主に健康相談室来室者、九州大学芸術工学院学生などの参加を想定している。

4.評価後のコンテンツやインターフェースの問題点(例えばアプリ内の某機能は全く使用されていない、興味を引かないなど)を改善し、次年度の実証実験用のアプリとして完成度を上げる。

介入方法: 2018年度以降は、当センターを利用している学生を対象に、本アプリケーションがメンタルヘルスの向上に有用か否かを検討する。また当センター利用者以外(授業などを介してリクルート)にも使用をすすめ、その前後での精神状態等の評価を行う。具体的には以下のような評価方法を考えている。

1.アプリケーション利用前後で精神状態を比較: 学生を対象として、アプリケーション利用前後の精神状態を比較する。アプリケーション利用期間は2週間~1ヶ月と設定し、評価方法はGHQ-12 (12項目からなる精神健康度調査票), CES-D (うつ病評価スケール)などを用いて、精神症状の改善または増悪の程度を評価する。

2.アプリケーション利用前後で、精神疾患に関する偏見やスティグマを比較: 対象学生にLinkのスティグマ尺度*に基づくアンケートをアプリケーション利用の前後で行い、精神疾患に対する考え(偏見など)をスコアリングする (*12項目からなるスティグマ評価尺度で、各設問は1点 (全くそう思わない)から4点 (非常にそう思う)の4段階で評価し、その合計点で精神疾患に対する偏見を検討する)。

3.アプリケーションの印象:アプリケーション利用後で、アプリケーションの印象についてたずねる。

以上のようにアプリケーションの効果を評価し、有効であれば更にアプリケーションの種類を増やして行きたい。

研究の期間: 2016年度~2021年度

評価項目: GHQ-12、CES-D、 Linkスティグマスケール。

データ解析の方法: 対象者の基本属性および介入前の各評価項目の群間比較には、名義変数の検定はカイ二乗検定またはFisherの正確確率検定を行い、連続変数の検定はShapiro-Wilkの正規性に基づき、t検定あるいはMann-Whitney U検定を行う。介入群と対照群の比較には、repeated measures ANOVAにて交互作用の有無を検討する。介入前後の差の検定には対応のあるt検定を行う。ただし、収集されたデータ結果によっては上記統計方法を必要に応じて変更することはある。

**対象者への倫理的配慮**

対象者に生じる利益と不利益: 謝金、経済的負担はない。身体的な介入ではないため、健康被害の可能性はない。

インフォームドコンセント: 本研究はメンタルヘルスに関するスコアリングの結果やアンケート結果を扱うため、個人情報の扱いには慎重を要する。研究に参加するボランティアに対しては試験研究について十分な説明を行い、インフォームドコンセントを得てから進める。結果は速やかに解析し、被験者に開示する。具体的な方法を以下に記す。

1. 得られた測定値などの個人情報はプライバシーを守る為に個人が特定できないようIDコード化し、個人情報の取り扱いには慎重を期する。
2. 研究の目的・方法・個人のリクスおよび利益等に関して文章、口頭で説明すると共に、研究への参加または中止（同意の撤回）は本人の自由であることや、秘密保持の保証等を文章・口頭で明示し、対象者より同意を得て同意書を作成し署名を得る。同意書は研究代表者が1部ずつ管理する。
3. 尚、研究対象者が未成年の場合は説明文書を2部配布し、1部は親権者等へ渡すよう伝え、オプトアウトの機会を保証する。同意書は、研究代表者自身あるいは研究代表者の監督のもとで研究分担者が施錠した保管庫に保存する。

研究データの二次利用(将来新たに計画・実施される研究へのデータの利用)についても説明し、同意を得られた場合のみ将来の研究へ活用する。

参加の任意性と同意撤回の自由の保障：上記の通り、インフォームドコンセントにおいて参加の任意性や撤回の自由について説明を口頭および文書で行う。

個人情報の保護: 個人情報は個人が特定されないIDを付与した後、収集データと分離して厳重に保管する。データは連結可能であるため対応表は研究代表者が施錠した保管庫にて責任を持って保管する。紙媒体は復元できないようシュレッダーで裁断し、電子データは消去ソフト等を用いて削除する。データの保存期間は当該論文発表後10年間とする。

**研究費と利益相反**

研究の財源として、科研費(16K13031)、QRプログラム、ヘルスサイエンスセンター研究助成金を使用する。本研究によって得られた全ての開示資料に対し利益相反に関係する者はいない。

**研究に関する情報公開**

研究結果は学会や学術論文にて公開する予定である。

**その他**

本研究は、2015年度の九州大学基幹教育院倫理委員会にて承認された内容であり、今回は研究期間延長のために再度申請したことを付言する。(前前回・前回倫理番号: 201508R・201819-1 )
