# Supplementary material for: Short-term effect of a smartphone application on the mental health of university students: A pilot study using a user-centered design self-monitoring application for mental health
Source: PLoS One. 2020 Sep 25;15(9):e0239592. doi: 10.1371/journal.pone.0239592 (PMC7518576; doi:10.1371/journal.pone.0239592)
Supplement: S2 File — (DOCX) [file pone.0239592.s004.docx]

**Research plan and protocol**

Application date: Dec 24^th^, 2015

Approval date :Feb 9^th^, 2016

Re-application date: Jan 19^th^, 2020

Re-approval date :Feb 20^th^, 2020

Kyushu University

Center for Health Sciences and Counseling

Kosuke Kajitani M.D. and Ph.D

**The title of research project**

Development and experimental study of mental health app for university students.

**Research team**

Principal investigator:

Kosuke Kajitan, Center for Health Sciences and Counseling, Kyushu University, 744, Motooka, Nishi-ku, Fukuoka, 816-8580, JAPAN, (tel-1)092-802-5116, (tel-2) 092-802-5124, e-mail: [kkajitani@chc.kyushu-u.ac.jp](mailto:kkajitani@chc.kyushu-u.ac.jp)

Co-investigators:

Ikumi Higashijima^1)^, Kosuke Kaneko^2)^, Tomoko Matsushita^3)^, Hideaki Fukumori^3)^,

Daewoong Kim^1)^, Rikako Tsuchimoto^3)^, Fumika Funatsu^3)^

^1^Department of Content and Creative Design, Faculty of Design, Kyushu University

^2^Cybersecurity Center, Kyushu University

^3^Center for Health Sciences and Counseling, Kyushu University

**Background of the study**

College days are when students are predisposed to get stressed from their studies in college, human relations. During this period, college students are physically mature but mentally immature, resulting “identity crisis” as Erikosn E.H. pointed out. The survey by the Ministry of Health, Labor and Welfare showed suicide to be the leading cause of death for Japanese in their 20s. Moreover, it has been reported that suicide victims may suffer from mental disorders such as depression and drug addiction (Arsenault-Lapierre, BMC Psychiatry, 2004). The applicant is engaged in consultation and treatment of students with mental health problems in Kyushu University. Surprisingly, the number of students who consulted about mental health problem has remained flat or declined for the past 10 years, while the suicide rate has been increasing in our university (unpublished data). Given that suicidal behavior is closely related to the presence of mental disorders, these results indicate that many students with mental health hesitate to consult with specialists including psychiatrist and counselors. In order to improve this situation, whereby university students seldom use consulting systems, we launched this project because smartphones are now an inextricable part of university students’ lives. In fact, more than 98% of university students own a smartphone in 2019. In this project, we planned to develop a smartphone app with the following three characteristics; 1) design familiar to students, 2) self-monitoring function for mental health, and 3) functions to promote self-care for students.

**The purpose of this study**

　The purposes of this study are as follows; 1) We independently develop a smartphone app called the ‘Mental App’, which is designed for university students according to the results obtained from a questionnaire and log data, 2) Using psychological tests, we examined the app’s effect on the mental state of university students. If this project were to go well, we would expect the following benefits to students; 1) increasing the consultation rate to specialist of mental health, 2) early detection of mental disorders and prevention of suicide, and 3) reducing stigma against mental disorders. These benefits will contribute not only to individual but to social well-being. Furthermore, if our app proves effective for mental health, it can be expected to adopt to other communities including workplace and nursing care for the elderly.

**Study design**

Study type**:** interventional

Basic design: parallel

Randomization: non-randomized

Data: We obtain new data (We will not receive data from other facilities).

Eligibility: Students in Kyushu University, who are 18 years of age and older. Exclusion criteria is those who cannot understand Japanese.

Recruitment: All participants were recruited from the University’s Interdisciplinary Graduate School of Engineering Sciences and the Faculty of Arts and Science. In the psychology class, we explained the study’s outline and asked for volunteers. Written informed consent was obtained from each participant after providing them with information about the study. The target number of participants is 100 based on the previous research (Ludtke, Psychiatry Res, 2018).

Method of the study:

Development of the smartphone application

In the first year of this study, we determine the content of the smartphone app. The prototype app consists of, 1) platform app (homepage of the app), 2) initial apps (the apps that come with the platform app when installed), 3) additional apps (users can add as needed). First, the user activates the ‘simple diagnosis application’ , which is one of the initial applications, to examine whether the user has mental disorders. The simple diagnosis application is developed with reference to the Mini-International Neuropsychiatric Interview (M.I.N.I.). According to the mental disorder diagnosed by the simple diagnosis application, the app recommends that the user install an additional app for a more detailed diagnosis. For example, if there is a possibility that the user has depression as a result of the simple diagnosis application, the app recommends the user to install an additional application based on Beck depression inventory (ISBN: 0-8122-1032-8). The additional app evaluates the severity of the mental disorder and advises coping methods depending on the severity. For example, in mild cases, the app will refer to self-care and campus counselors, and in severe and emergency cases, the app will refer to a nearby hospitals or clinic. However, the content of the app may be partially changed due to budget restrictions.

　In the second year of this study, we design the app for university students. To refine the design of the app, we develop the app by the following steps;

1. We investigate commercially available apps that are popular with university students and design the interfaces of the app.
2. Base on step 1, we develop a prototype app.
3. We ask students to use the prototype app. We look for something wrong with the app including program bugs and usability. Log data are collected to assess the prototype app usage, including the frequency and behavioral patterns. We also conduct a questionnaire survey on the overall design impression and usability of the app.
4. We improve all problems of the prototype app, and make the final version of the app.

Intervention with the final version of the app.

The final version of the app is installed on the students’ smartphone and the psychological tests (the Link Stigma Scale, the Center for Epidemiologic Studies Depression Scale, and the 12-item General Health Questionnaire) are conducted on the same day. After using the app for two or four weeks, the students complete the questionnaire survey and undergo the same psychological tests. We compare the results between the app user and non-user group.

Research period: Immediately after approved by the Ethics Committee until fiscal year 2021.

Primary outcome: Changes in the results of psychological tests (Link stigma scale, CES-D, GHQ-12) before and after using the app.

Data analysis: Chi-square or Fisher’s exact tests is used to compare the categorical variables between the groups. For the test of continuous variables, t-test or Mann-Whitney U test is performed based on the normality of Shapiro-Wilk test. For comparison between intervention and control group, the repeated measures ANOVA is used to examine the interaction. A paired t-test is used to test the difference before and after the intervention. However, the statistical method may be changed depending on the collected data.

**Ethical consideration for subjects**

Benefit and risk: There are no rewards in this study. Since it is not a physical intervention, there is no potential health hazard.

Informed consent: Since this study deals with score and questionnaire regarding mental health, it is necessary to handle personal information with caution. We will fully explain the outline of study to participants and obtain informed consent in advance. The results will be analyzed and disclosed to participants as soon as possible. The procedure is described as follows;

1. To protect privacy of participants, the obtained personal information is encoded with ID number so that it cannot be identified as the person.
2. We explain the purpose, method, risk and benefits of the study to the subjects. We obtain written informed consent from the subject. We clearly state that subject is free participate of decline the study (withdrawal of consent). Furthermore, we guarantee the confidentiality of the personal information obtained in this study. After explaining the study, we ask the subject to sign a consent form. The written consent is retained by the principal investigator. We also explain the secondary use of research data and use it for future study only if consent is obtained.
3. If the participants are under the age of 20, we will provide two copies of the form explaining the study and ask them to give the form to their parents to ensure opt-out opportunities.

Protection of personal information: Personal information should be kept strictly separate from collected data. Since the obtained data can be linked with personal information, the corresponding table will be stored in the storage locked by the principal investigator. The paper medium is shredded so that it cannot be restored, and the electronic data is deleted using data erasing software. Data will be retained for 10 years after the publication of the study.

**Funding:** This study was supported by grants from the Japan Society for the Promotion of Science KAKENHI Grant Number 16K13031 (to K. Kajitani), Health Science Center Foundation (to K. Kajitani), and Qdai-jump Research Program Grant Number 28314 (to K. Kajitani).

**Competing interests:** We declare that there are no competing interests.

**Information disclosure about this study:** This research results will be published at academic conferences and academic journal.

**Note:** This research was approved by the Ethics Committee of the Faculty of Arts and Science and Center for Health Sciences and Counseling at Kyushu University in 2015, and we applied again for the extension of the research period (application number: 201508R and 201819-1).
