# Supplementary material for: Short-term effect of a smartphone application on the mental health of university students: A pilot study using a user-centered design self-monitoring application for mental health
Source: PLoS One. 2020 Sep 25;15(9):e0239592. doi: 10.1371/journal.pone.0239592 (PMC7518576; doi:10.1371/journal.pone.0239592)
Supplement: Peer review history

# Peer review: Decision Letter 0

**DOI:** 10.1371/journal.pone.0239592.r001

**Authors:** Kenji Hashimoto

10 Jun 2020

PONE-D-20-11603

Short-term effect of a smartphone application on the mental health of university students: A pilot study using a user-centered design self-monitoring application for mental health

PLOS ONE

Dear Dr. Kajitani,

Thank you for submitting your manuscript to PLOS ONE. After careful consideration, we feel that it has merit but does not fully meet PLOS ONE’s publication criteria as it currently stands. Therefore, we invite you to submit a revised version of the manuscript that addresses the points raised during the review process.

The reviewers addressed several major and minor concerns about your manuscript. Please revise the manuscript carefully.

Please submit your revised manuscript by Jul 25 2020 11:59PM. If you will need more time than this to complete your revisions, please reply to this message or contact the journal office at plosone@plos.org. When you're ready to submit your revision, log on to https://www.editorialmanager.com/pone/ and select the 'Submissions Needing Revision' folder to locate your manuscript file.

Please include the following items when submitting your revised manuscript:

- A rebuttal letter that responds to each point raised by the academic editor and reviewer(s). You should upload this letter as a separate file labeled 'Response to Reviewers'.
- A marked-up copy of your manuscript that highlights changes made to the original version. You should upload this as a separate file labeled 'Revised Manuscript with Track Changes'.
- An unmarked version of your revised paper without tracked changes. You should upload this as a separate file labeled 'Manuscript'.

If you would like to make changes to your financial disclosure, please include your updated statement in your cover letter. Guidelines for resubmitting your figure files are available below the reviewer comments at the end of this letter.

If applicable, we recommend that you deposit your laboratory protocols in protocols.io to enhance the reproducibility of your results. Protocols.io assigns your protocol its own identifier (DOI) so that it can be cited independently in the future. For instructions see: http://journals.plos.org/plosone/s/submission-guidelines#loc-laboratory-protocols

We look forward to receiving your revised manuscript.

Kind regards,

Kenji Hashimoto, PhD

Academic Editor

PLOS ONE

Journal Requirements:

When submitting your revision, we need you to address these additional requirements.

1. Please ensure that your manuscript meets PLOS ONE's style requirements, including those for file naming. The PLOS ONE style templates can be found at https://journals.plos.org/plosone/s/file?id=wjVg/PLOSOne_formatting_sample_main_body.pdf and https://journals.plos.org/plosone/s/file?id=ba62/PLOSOne_formatting_sample_title_authors_affiliations.pdf

2. Please include captions for your Supporting Information files at the end of your manuscript, and update any in-text citations to match accordingly. Please see our Supporting Information guidelines for more information: http://journals.plos.org/plosone/s/supporting-information.

Additional Editor Comments (if provided):

[Note: HTML markup is below. Please do not edit.]

Reviewers' comments:

Reviewer's Responses to Questions

**Comments to the Author**

1. Is the manuscript technically sound, and do the data support the conclusions?

The manuscript must describe a technically sound piece of scientific research with data that supports the conclusions. Experiments must have been conducted rigorously, with appropriate controls, replication, and sample sizes. The conclusions must be drawn appropriately based on the data presented. 

Reviewer #1: No

Reviewer #2: No

Reviewer #3: Yes

**********

2. Has the statistical analysis been performed appropriately and rigorously? 

Reviewer #1: No

Reviewer #2: N/A

Reviewer #3: Yes

**********

3. Have the authors made all data underlying the findings in their manuscript fully available?

The PLOS Data policy requires authors to make all data underlying the findings described in their manuscript fully available without restriction, with rare exception (please refer to the Data Availability Statement in the manuscript PDF file). The data should be provided as part of the manuscript or its supporting information, or deposited to a public repository. For example, in addition to summary statistics, the data points behind means, medians and variance measures should be available. If there are restrictions on publicly sharing data—e.g. participant privacy or use of data from a third party—those must be specified.

Reviewer #1: No

Reviewer #2: Yes

Reviewer #3: Yes

**********

4. Is the manuscript presented in an intelligible fashion and written in standard English?

PLOS ONE does not copyedit accepted manuscripts, so the language in submitted articles must be clear, correct, and unambiguous. Any typographical or grammatical errors should be corrected at revision, so please note any specific errors here.

Reviewer #1: Yes

Reviewer #2: No

Reviewer #3: Yes

**********

5. Review Comments to the Author

Please use the space provided to explain your answers to the questions above. You may also include additional comments for the author, including concerns about dual publication, research ethics, or publication ethics. (Please upload your review as an attachment if it exceeds 20,000 characters)

Reviewer #1: Dear authors

This manuscript evaluates the effect of a mental health training app based on self monitoring on stigma and mental health. It is overall well written, although it could be shorter. I have a major concern on how the analysis was done and some minor points.

1. Abstract: please ensure that special signs (delta) are permitted for the abstract and will be correctly displayed in the final abstract. This is not the case in the current abstract on the first page of the PDF.

2. Line 195: I do not see the need to report on which computer models the app was developed. Similarly, on line 208, the details about the storage server are of little interest.

3. Results: p is usually written in lowercase. p=0.0000 is usually reported as p<.001 (line 294)

4. Line 451: The user group did improve in “mental health”, not “mental health care”.

5. Line 165: was the self diagnosis simply “do you have this disorder” or was there a multi-item screener?

6. Why are the delta displayed in figure 4 and not the actual means? I do not see much use in that. Especially as the delta in the pre-study is simply 0 in all figures. This masks the fact that the groups were different (almost significant at p=0.056) on the GHS score at the pre-test, but not at the post-test (p=0.826), and that the app did only rebalance initial differences between the groups. Related to this, one major limitation is regression to the mean: those with a higher initial scores (thus in the app user group) are more likely to decrease towards the mean and have more potential to change. I believe that simply comparing difference scores is insufficient as analysis. A method that adjusts for initial values (i.e. some kind of baseline adjustment) is needed. There are many standard regression approaches that can do this. I believe that there is a solid chance that the difference in delta is not significant if adjusted for the initial GHS score and the app has therefore no effect at all.

7. Line 315: why do you speak of variation here, when the delta appear to be a simple difference score. Variation is reserved for variance in my opinion.

8. Line 320: How is the variability lower in the app user group? The difference is of far larger magnitude (albeit more negative and therefore lower if you want to put it like that) in the app user group.

Reviewer #2: Mental health is an issue of importance for human happiness. Also, utilizing information technology to promote mental health of citizens is a modern theme of psychiatry. I myself is interested in the theme of this manuscript very much.

I have to say, however, that this study is far from satisfactory as a responsible clinical trial.

First of all, the authors mentioned that they could not foresee any potential safety issue. I hardly understand it. The authors believe that using the app would promote participant's mental health, doesn't they? Why did the authors deem this study not as a clinical trial? Why did the ethics committee miss the fact before approving the protocol? I have to say that the process of implementing the study includes a critical flaw.

The authors honestly mentioned that they registered this study on UMIN clinical trials registry. Delaying of the registration is not a critical issue.

On the other hand, did the researchers explain the risk of adverse effects with participating this study to the participants in advance? If not, this study is inappropriately conducted, I cannot accept the publishing.

The authors wrote that participants were divided into an app user group and a non-user group. And this study is not randomized controlled trial. How were they divided? An arbitrarily allocation of the groups decline the value of study much. The authors have to clarify the way of allocation concretely.

The authors wrote that using the app participants could self-diagnosis from 14 mental disorders including schizophrenia. I hardly believe it. Determining clinical diagnosis of mental disorders is not an easy task even for a skilled psychiatrist. Even latest criteria (DSM-5 and ICD-11) do not accomplish acceptable level of reliability. As far as reading this manuscript, the range of data of each participant input to the app is very limited. For example, up to 30% of depressive patients excessively eat meal. Also, a half of patients with depression never express depressive mood. Overall, it is far from my understanding that the authors sincerely believe patients can diagnose their mental disorders with this app.

The app shows nearest clinic for participants, according to the manuscript. Showing a contact number of a school counselling office may have no problem. But, referring a specific clinic can be deemed as a medical advice. Are the clinics involved in the study? If so, they are potentially conflict of interest. If not, can the developer of the app take the responsibility when some accidents occur in a clinic referred.

The participants answered LSS questionnaire twice in this study in a couple of week. Can LSS be used repeatedly such a short interval? I guess many participants remembered their past answers which influenced the result of the post test.

Overly, I suspect that the authors misunderstand the concept of clinical trial as well as mental disorders. A skilled supervisor should have been involved in the stage of planning the grand design of the whole research.

Minor comments and suggestions:

One of the barriers for students from consulting a medical practitioner may be financial issue. In recent, many university students have economical problems. This kind of apps will potentially be an aid for poor users. In this sense, I recommend the authors to develop the app of Android version because Android smartphones are generally not costly than iPhone, as the authors may know.

Why did the author limit the users of the app to university students? Was this app optimized for university students (e.g., mentioning learning issue, screening mild developmental disorders)? As the age of university students are various, it is better to estimate over 40s users. But, points of issue for elder students are different from those of late adolescents. I recommend to care for this issue in the future development.

Reviewer #3: The authors developed a smartphone application (Mental App) for the university

students and examined the effects of the app on their mental health. They found a significant between-group difference in the General Health

Questionnaire score. The findings will be of interest to researchers in the field.

I have the following concerns.

#1 Materials and methods, P8 line 120, “We did not register this study as a clinical trial before the enrolment of participants started, because we could not foresee any potential safety issue. We registered this study retrospective at UMIN clinical trials registry (UMIN000040332) after we were reminded that our study design indeed met the WHO’s definition of a clinical trial.”

I think whether or not this is permitted depends on the PLOS One policy, not on the reviewer’s private opinion. I would like to leave a decision to PLOS One.

#2. Table 3. Generally, the cut-off point for common mental disorders is 15/16 for CES-D and 2/3 for GHQ-12. However, Table 3 shows high mean test scores (CES-D score: 16.2 ± 12.1 vs. 15.8 ± 11.2, GHQ-12 score: 3.10± 2.85 vs. 2.93 ± 3.37), suggesting that more than half of subjects were beyond the cut-off points. Please discuss this point.

In conclusion, I enjoyed reading this paper. These findings will be of interest to practitioners, as well as researchers in the field.

**********

6. PLOS authors have the option to publish the peer review history of their article (what does this mean?). If published, this will include your full peer review and any attached files.

If you choose “no”, your identity will remain anonymous but your review may still be made public.

**Do you want your identity to be public for this peer review?** For information about this choice, including consent withdrawal, please see our Privacy Policy.

Reviewer #1: No

Reviewer #2: Yes: Akihiro Shiina

Reviewer #3: No

[NOTE: If reviewer comments were submitted as an attachment file, they will be attached to this email and accessible via the submission site. Please log into your account, locate the manuscript record, and check for the action link "View Attachments". If this link does not appear, there are no attachment files.]

While revising your submission, please upload your figure files to the Preflight Analysis and Conversion Engine (PACE) digital diagnostic tool, https://pacev2.apexcovantage.com/. PACE helps ensure that figures meet PLOS requirements. To use PACE, you must first register as a user. Registration is free. Then, login and navigate to the UPLOAD tab, where you will find detailed instructions on how to use the tool. If you encounter any issues or have any questions when using PACE, please email PLOS at figures@plos.org. Please note that Supporting Information files do not need this step.

# Author comment: Author response to Decision Letter 0

**DOI:** 10.1371/journal.pone.0239592.r002

30 Aug 2020

Responses to reviewer comments

We thank the reviewers for their valuable comments and suggestions regarding our manuscript. We have revised our manuscript accordingly. The changes made in response to the reviewers’ comments are marked in red in the revised manuscript and this rebuttal.

Responses to the comments of Reviewer #1

Remarks to the Author

Reviewer #1:

This manuscript evaluates the effect of a mental health training app based on self- monitoring on stigma and mental health. It is overall well written, although it could be shorter. I have a major concern on how the analysis was done and some minor points.

1. Abstract: please ensure that special signs (delta) are permitted for the abstract and will be correctly displayed in the final abstract. This is not the case in the current abstract on the first page of the PDF.

Author’s Response: Thank you for your comment. Following your advice, we have corrected the font by use of “Unicode".

(page 4, line 45: abstract)

There were no significant between-group differences in the ΔLink Stigma Scale score (-0.11 ± 4.28 vs. -0.59 ± 3.30, p = 0.496) or the ΔCenter for Epidemiologic Studies Depression Scale score (-4.39 ± 7.13 vs. -2.07 ± 8.78, p = 0.143). There was a significant between-group difference in the ΔGeneral Health Questionnaire score (-2.21± 2.23 vs. -0.17 ± 2.69, p = 0.007).

2. Line 195: I do not see the need to report on which computer models the app was developed. Similarly, on line 208, the details about the storage server are of little interest.

Author’s Response: We appreciate and agree with the reviewer’s suggestion. Accordingly, we have removed the text outlined. 

(page 13, line 200) (page 14, line 210)

3. Results: p is usually written in lowercase. p=0.0000 is usually reported as p<.001 (line 294)

Author’s Response: Thank you for your comment. Following the reviewer’s advice, we have corrected this to “p < .001”.

(page 20, line 296)

...that in the post-study score (4.86 ± 3.54 vs. 2.64 ± 3.00, p < .001).

In addition, we have updated the uppercase “P”s to lowercase “p”s in this manuscript.

(page 4, line 46-48 ), (page 17, line 255 ), (page 19, line 286-290), (page 20, line 294-300), (page 21, line 306-307) , (page 21, line 312-315), (page 21, line 320-323 ), (Table 1), and (Table 3).

4. Line 451: The user group did improve in “mental health”, not “mental health care”.

Author’s Response: We apologize for this error. We have corrected this to “mental health”.

(page 32, line 491-492)

...could improve mental health in university students.

5. Line 165: was the self-diagnosis simply “do you have this disorder” or was there a multi-item screener?

Author’s Response: Thank you for this important comment. The self-diagnosis system of the Mental App is based on a multi-item screener. For example, the Mental App asks the users whether the users have felt depressed over the past 2 weeks, whether they have been less interested in any activities, whether they can sleep enough, etc… for the diagnosis of depression. To clarify this point, we have added the sentences as follows;

(Page 12, line 173-174):

The self-screening for mental disorders was based on the multi-item measures of each mental disorder.

6. Why are the delta displayed in figure 4 and not the actual means? I do not see much use in that. Especially as the delta in the pre-study is simply 0 in all figures. This masks the fact that the groups were different (almost significant at p=0.056) on the GHS score at the pre-test, but not at the post-test (p=0.826), and that the app did only rebalance initial differences between the groups. Related to this, one major limitation is regression to the mean: those with a higher initial scores (thus in the app user group) are more likely to decrease towards the mean and have more potential to change. I believe that simply comparing difference scores is insufficient as analysis. A method that adjusts for initial values (i.e. some kind of baseline adjustment) is needed. There are many standard regression approaches that can do this. I believe that there is a solid chance that the difference in delta is not significant if adjusted for the initial GHS score and the app has therefore no effect at all.

Author’s Response: Thank you for this important insight. To compare the changes in the measured values, the delta has been used in some fields of medicine such as physiology (e.g., change of blood pressure). However, as you pointed out, a regression to the mean is an issue when comparing the change from baseline between the two groups. To solve this problem, we conducted the univariate ANCOVA to analyze the score change (post - pre) in the independent variables by the group (app user or non-user) with the pre-test scores as covariates. The ANCOVA analysis found a statistically significant difference [F (1, 54) = 5.72, p = 0.020] between the app-user and non-user group in GHQ-12. However, there were no significant differences between the app-user and non-user group in the LSS [F (1, 54) = 0.11, p = 0.74] and CES-D [F (1, 54 ) = 0.12, p = 0.74]. Accordingly, we have added the following sentences and the table (as Table 4) to the manuscript;

(page 17, line 253: Material and Method) 

We conducted the univariate ANCOVAs to test for differences between the groups (app user and non-user), with pre-test values as the covariate.

(page 22, line 325: Results):

To exclude the possibility that the difference in the ΔGHQ was caused by a regression to the mean, univariate ANCOVA was used to assess the effect of using the Mental App. The ANCOVA analysis found a statistically significant difference [F (1, 54) = 5.72, p = 0.020] between the app user and non-user group in GHQ-12. However, there were no significant differences between the app user and non-user group in the LSS [F (1, 54) = 0.11, p = 0.74] and CES-D [F (1, 54) = 0.12, p = 0.74] (Table 4).

(Page 22, line 332: please see Table 4)

Table 4. Comparison of app user and non-user group using ANCOVAs in post-test with pre-test as covariates

7. Line 315: why do you speak of variation here, when the delta appear to be a simple difference score. Variation is reserved for variance in my opinion.

Author’s Response: Thank you for your comment. We compared "the amount of change between pre- and post-intervention” between the app-user and non-user group here. Therefore, we have corrected this part as follows;

(page 21, line 317)

Fig 4. Comparison of the difference in the psychological test scores before and ...

8. Line 320: How is the variability lower in the app user group? The difference is of far larger magnitude (albeit more negative and therefore lower if you want to put it like that) in the app user group.

Author’s Response: Thank you for your comment and we apologize, this was a grammatical mistake. We meant that ΔGHQ-12 of the app-user group was significantly greater than that of the non-user group. We have corrected the sentences as follows;

(page 22, line 322)

The app user group showed a significantly greater difference score than that of the non-user group (p = 0.007).

 

Responses to the comments of Reviewer #2

Remarks to the Author

Reviewer #2:

Mental health is an issue of importance for human happiness. Also, utilizing information technology to promote mental health of citizens is a modern theme of psychiatry. I myself is interested in the theme of this manuscript very much. I have to say, however, that this study is far from satisfactory as a responsible clinical trial.

1. First of all, the authors mentioned that they could not foresee any potential safety issue. I hardly understand it. The authors believe that using the app would promote participant's mental health, doesn't they? Why did the authors deem this study not as a clinical trial? Why did the ethics committee miss the fact before approving the protocol? I have to say that the process of implementing the study includes a critical flaw. The authors honestly mentioned that they registered this study on UMIN clinical trials registry. Delaying of the registration is not a critical issue. On the other hand, did the researchers explain the risk of adverse effects with participating this study to the participants in advance? If not, this study is inappropriately conducted, I cannot accept the publishing.

Author’s Response: Thank you for these important comments. We genuinely thought that this pilot-study did not need a clinical registration as it is not a physically invasive study and we did not foresee any potential safety issues including health problems for the subjects (Participants just used the app and answered the psychological tests). We explained the outline of the study to participants and obtained informed consent (IC) in advance. In the IC, we clearly stated that they could freely withdraw the study (discontinue participation). Furthermore, many other interventional studies, which examined the effect of smartphone apps on mental health, did not mention the safety issues nor the registration of the studies as a clinical trial in their reports, although they did obtain IC from the participants (Bakker et al., J Affect Dis, 2018; Carey et al., BMC Psychiatry, 2016; Geyer et al., Behavior Therapy, 2018; Kenny et al., JMIR Mental Health, 2015; Ludtke et al., Psychiatry Res, 2018). In addition, our Ethics Committee did not point out any potential safety issue of this study. For these reasons, this study was retrospectively registered as a clinical study after the editorial staff of PLOS ONE pointed out the need for registration. We have added the sentence to explain the reason why we registered this study as follows;

(page 8, line 120):

We did not register this study as a clinical trial before the enrolment of participants started, as we could not foresee any potential safety issues including any health problems. The Ethics Committee did not identify any safety risks and therefore did not require this study to be registered as a clinical trial.

Furthermore, the PLOS ONE states that they consider publishing the retrospectively registered clinical trials if the studies meet the criteria of PLOS ONE guideline. We believe that our study meets these criteria (https://everyone.plos.org/2013/10/21/plos-one-supports-registration-and-reporting-of-all-trials/).We leave this issue to the discretion of the editor.

However, reviewer #2 has raised an important point regarding ethics of research and we will keep it in mind for future studies. Thank you for your advice.

2.The authors wrote that participants were divided into an app user group and a non-user group. And this study is not randomized controlled trial. How were they divided? An arbitrarily allocation of the groups decline the value of study much. The authors have to clarify the way of allocation concretely.

Author’s Response: Thank you for raising this point. As described in the report, this pilot-study is a non-randomized test. In the psychology class, we explained the study’s outline and asked for volunteers. When explaining the study, we also told the students that “If you are an iPhone user, you can install and use this app. On the other hand, you can join the experiment as a control group (non-user) if you do not want to use the app or if you are an Android user”. Many subjects of the non-user group were iPhone user but there were several Android users as well. In summary, the subjects were allocated following their free will to join whichever group. To clarify this, we have added the sentence as follows;

(page 9, line 129)

It was up to the subjects to decide whether they wanted to join the app user or the non-user group.

Needless to say, we already discussed this point as a limitation of study.

(page 31, line 466)

The present study has some limitations. First, this study was not an RCT, so the results may have been affected by the potential confounding factors. In our study, only iPhone users could participate in the intervention group (app user group) and other types of mobile phone users were allocated to the control group (non-user group).

3.The authors wrote that using the app participants could self-diagnosis from 14 mental disorders including schizophrenia. I hardly believe it. Determining clinical diagnosis of mental disorders is not an easy task even for a skilled psychiatrist. Even latest criteria (DSM-5 and ICD-11) do not accomplish acceptable level of reliability. As far as reading this manuscript, the range of data of each participant input to the app is very limited. For example, up to 30% of depressive patients excessively eat meal. Also, a half of patients with depression never express depressive mood. Overall, it is far from my understanding that the authors sincerely believe patients can diagnose their mental disorders with this app.

Author’s Response: We appreciate and agree with the reviewer’s suggestion. We admit that determining a clinical diagnosis of mental disorders is difficult. However, this app only implies the possibility of a mental disorder to the subjects. The diagnosis function of this app is a simple self-reported screening test. Since the term “self-diagnosis” appears to cause a misunderstanding, we have updated “self-diagnosis" to “self-screening for mental disorders” throughout the manuscript.

In addition, to screen for schizophrenia, we used the PRIME screen (Japanese version) which is a self-administered questionnaire designed to quickly assess the risk for developing a psychotic disorder (We obtained the permission to use the PRIME screen from Professor Dr. Masafumi Mizuno of Department of Neuropsychiatry at the Toho University). Strictly speaking, this is a screening test for the risk of psychosis rather than schizophrenia. Therefore, we have updated the description from schizophrenia to psychotic states.

(page 8, line 110)

This user-centered designed app was equipped with a self-monitoring and self-screening function for mental disorders aimed to tackle the aforementioned obstacles...

(page 11, line 161)

...1) a self-monitoring function, 2) a self-screening function for mental disorders, ...

(page 12, line 169)

In the self-screening function for mental disorders, participants could conduct a brief screening for..

(page 12, line 173)

The self-screening for mental disorders was based on the multi-item measures of each mental disorder. According to...

(page 13, line 191)

(E) The main screen of the screening function for mental disorders. Users…

(page 18, line 276)

 … and 23 out of the 35 subjects used the self-screening function (Table 2).

(page 19, Table 2)

Use of the ‘self-screening function’ (yes/no)

(page 24, line 349)

self-screening function (14 mental disorders) and a referral function ...

(page 29, line 437)

The app had a self-screening function for mental disorders that provided general information…

(page 29, line 437)

…our app did not prompt students to use the self-screening function and ...

(Fig 2E)

Diagnosis => Screening

(page 12, line 171)

…, psychotic state, generalized anxiety disorder, panic disorder,

4.The app shows nearest clinic for participants, according to the manuscript. Showing a contact number of a school counselling office may have no problem. But, referring a specific clinic can be deemed as a medical advice. Are the clinics involved in the study? If so, they are potentially conflict of interest. If not, can the developer of the app take the responsibility when some accidents occur in a clinic referred.

Author’s Response: Thank you for your comment. Our application is linked with the "Google Maps" and participants can check the contact information of nearby clinics, as publicly listed by Google Maps. Therefore, there is no COI to disclose. To further clarify this point, we have added the sentences as follows in material and methods;

(page 12, line 177):

Users could search the location and contact information provided by “Google Maps”, linked within the Mental App.

5.The participants answered LSS questionnaire twice in this study in a couple of week. Can LSS be used repeatedly such a short interval? I guess many participants remembered their past answers which influenced the result of the post test.

Author’s Response: We appreciate and agree with the reviewer’s comment. In the limitation section of this study, we mentioned that the duration of this study was too short to evaluate the efficacy of the Mental App. We may have observed a larger anti-stigma effect with a longer intervention time. We direct your attention to the following section:

(page 32, line 477):

Third, the duration of this study was too short to evaluate the efficacy of the Mental App. A longer observation period may reveal additional benefits or even adverse effects of our app. In particular, we think that a longer intervention time may be required for university students to experience changes in the stigma.

6.Overly, I suspect that the authors misunderstand the concept of clinical trial as well as mental disorders. A skilled supervisor should have been involved in the stage of planning the grand design of the whole research.

Author’s Response: Thank you for your comment. We will consult a skilled supervisor for our further studies.

7.One of the barriers for students from consulting a medical practitioner may be financial issue. In recent, many university students have economical problems. This kind of apps will potentially be an aid for poor users. In this sense, I recommend the authors to develop the app of Android version because Android smartphones are generally not costly than iPhone, as the authors may know.

Author’s Response: We appreciate and agree with the reviewer’s suggestion. We initially planned to develop an Android version of the app as well; however, we did not have the necessary budget to develop the Android version. For the future, we would like to develop an Android version of the Mental App and like to distribute this app to all university students. We have added the sentence as follows:

(page 31, line 473):

In the future, we would like to create an Android version of the Mental App to confirm the effect of the app on mental health in students and distribute this app to the university students of various backgrounds.

8.Why did the author limit the users of the app to university students? Was this app optimized for university students (e.g., mentioning learning issue, screening mild developmental disorders)? As the age of university students are various, it is better to estimate over 40s users. But, points of issue for elder students are different from those of late adolescents. I recommend to care for this issue in the future development.

Author’s Response: Thank you for this suggestion. We work in campus clinics to support the mental health of university students. This is the main reason why we developed the App for university students. Regarding the optimization for users, we optimized the design of the app for university students based on the results of a questionnaire survey, completed prior to this study.

The Mental App did not include a function to evaluate the academic problems such as the learning issue and screening mild developmental disorders. In addition, we agree with your suggestion that we should pay more attention to the elder students. In the future, we would like to expand the function of the Mental App to deal with these issues. Therefore, we have added the sentence to the limitation of this study as follows;

(page 32, line 481):

Finally, the Mental App did not include any function that dealt with academic problems (e.g., academic performance, mild developmental disorders, harassment, and employment after graduating the university). In addition, students of various ages, including middle-aged graduate students, also attend the university. Therefore, it is necessary to further expand the app’s functions to encompass such additional problems and diverse users at the university.

 

Responses to the comments of Reviewer #3

Remarks to the Author

Reviewer #3:

The authors developed a smartphone application (Mental App) for the university students and examined the effects of the app on their mental health. They found a significant between-group difference in the General Health Questionnaire score. The findings will be of interest to researchers in the field. I have the following concerns.

#1 Materials and methods, P8 line 120, “We did not register this study as a clinical trial before the enrolment of participants started, because we could not foresee any potential safety issue. We registered this study retrospective at UMIN clinical trials registry (UMIN000040332) after we were reminded that our study design indeed met the WHO’s definition of a clinical trial.”

I think whether or not this is permitted depends on the PLOS One policy, not on the reviewer’s private opinion. I would like to leave a decision to PLOS One.

Author’s Response: Thank you for your neutral and fair opinion. We agree and would like to leave this issue to the editor’s discretion. We genuinely thought that this pilot-study did not need a clinical registration as it is not a physically invasive study and we did not foresee any potential safety issues including health problems for the subjects (Participants just used the app and answered the psychological tests). We explained the outline of the study to participants and obtained informed consent (IC) in advance. In the IC, we clearly stated that they could freely withdraw the study (discontinue participation). Furthermore, many other interventional studies, which examined the effect of smartphone apps on mental health, did not mention the safety issues nor the registration of the studies as a clinical trial in their reports, although they did obtain IC from the participants (Bakker et al., J Affect Dis, 2018; Carey et al., BMC Psychiatry, 2016; Geyer et al., Behavior Therapy, 2018; Kenny et al., JMIR Mental Health, 2015; Ludtke et al., Psychiatry Res, 2018). In addition, our Ethics Committee did not point out any potential safety issue of this study. For these reasons, this study was retrospectively registered as a clinical study after the editorial staff of PLOS ONE pointed out the need for registration. Therefore, we have added the sentence to explain the reason why we registered this study as follows;

(page 8, line 120):

We did not register this study as a clinical trial before the enrolment of participants started, as we could not foresee any potential safety issues including any health problems. The Ethics Committee did not identify any safety risks and therefore did not require this study to be registered as a clinical trial.

For future studies, we take note to register this kind of study as a clinical trial.

#2. Table 3. Generally, the cut-off point for common mental disorders is 15/16 for CES-D and 2/3 for GHQ-12. However, Table 3 shows high mean test scores (CES-D score: 16.2 ± 12.1 vs. 15.8 ± 11.2, GHQ-12 score: 3.10± 2.85 vs. 2.93 ± 3.37), suggesting that more than half of subjects were beyond the cut-off points. Please discuss this point.

Author’s Response: Thank you for your important suggestion. As you pointed out, the mean score of CES-D and GHQ-12 seemed to be high in our study. We think that there two reasons for this discrepancy. First, subjects may have included students with mental health issues. The volunteer subjects may have participated in the study as they were concerned for their mental health. Therefore, it is possible that students with mental health problems may have raised the mean score of these psychological tests. Second, the cultural background in Japan may have impacted the results. Several studies show a relatively high score of these psychological tests in Japan. For example, Doi Y et al. performed GHQ-12 for the Japanese general adult population (1,808 subjects) and found that young adults (20-29 years) exhibited relatively high scores compared to the elders (men: 2.77± 2.86, women: 3.88± 3.16)(Doi, Y, PCN, 2003, 57: 379-383). Ohtsu et al. evaluated the mental health status of 1,619 medical students and found the mean (± SD) score of male students to be 3.00 (± 2.94) and that of female students to be 3.82 (± 3.10) (Ohtsu T, Acta Medica Okayama, 2014, 68:331-337). Concerning CES-D, a previous study examined the prevalence of depression among 2,200 Japanese people using CES-D and found the mean (± SD) CES-D score to be 16.09 (± 8.61) (Fushimi, Community Meant Health J, 2013). Furthermore, another study in Japan showed that 60.1 % of college students exceeded the cut-off point of CES-D (≥ 16). Therefore, the psychological tests that examine mental problems may tend to score relatively higher in Japan.

Accordingly, we have added text to the discussion section to explain as to why the scores of CES-D and GHQ-12 were relatively high in our study as follows;

(page 28, line 418)　

In general, the cut-off point for CES-D is 15/16 and that for GHQ-12 is 2/3; however, the mean scores of these tests were relatively high in our studies. There may be two reasons for this discrepancy. First, all subjects in the present study were student volunteers and some students with mental health problems may have participated in the study. Students with mental health problems may have been interested in our research due to concerns regarding their psychological state. Therefore, students with mental health problems may have raised the mean score of these psychological tests. Second, cultural background in Japan may have also further impacted the results of this study. Several studies report a relatively high score of CES-D and GHQ-12 in Japan. For example, Doi et al. performed GHQ-12 on the Japanese general adult population (1,808 subjects) and found that young adults (20-29 years) exhibited relatively high scores compared to the elders (men: 2.77± 2.86, women: 3.88± 3.16) [32]. Ohtsu et al. evaluated the mental health status of 1,619 medical students and found the mean (± SD) score of male students to be 3.00 (± 2.94) and that of female students to be 3.82 (± 3.10) [46]. Concerning CES-D, a previous study examined the prevalence of depression among 2,200 Japanese people using CES-D and found the mean (± SD) CES-D score to be 16.09 (± 8.61) [47]. Furthermore, another study in Japan showed that 60.1 % of college students exceeded the cut-off point of CES-D (≥ 16) [48]. Therefore, the psychological tests that examine mental problems may tend to score relatively higher in Japan.

Attachment Submitted filename: Response_to_Reviewers.docx Click here for additional data file.

# Peer review: Decision Letter 1

**DOI:** 10.1371/journal.pone.0239592.r003

**Authors:** Kenji Hashimoto

10 Sep 2020

Short-term effect of a smartphone application on the mental health of university students:A pilot study using a user-centered design self-monitoring application for mental health

PONE-D-20-11603R1

Dear Dr. Kajitani,

We’re pleased to inform you that your manuscript has been judged scientifically suitable for publication and will be formally accepted for publication once it meets all outstanding technical requirements.

Within one week, you’ll receive an e-mail detailing the required amendments. When these have been addressed, you’ll receive a formal acceptance letter and your manuscript will be scheduled for publication.

An invoice for payment will follow shortly after the formal acceptance. To ensure an efficient process, please log into Editorial Manager at http://www.editorialmanager.com/pone/, click the 'Update My Information' link at the top of the page, and double check that your user information is up-to-date. If you have any billing related questions, please contact our Author Billing department directly at authorbilling@plos.org.

If your institution or institutions have a press office, please notify them about your upcoming paper to help maximize its impact. If they’ll be preparing press materials, please inform our press team as soon as possible -- no later than 48 hours after receiving the formal acceptance. Your manuscript will remain under strict press embargo until 2 pm Eastern Time on the date of publication. For more information, please contact onepress@plos.org.

Kind regards,

Kenji Hashimoto, PhD

Section Editor

PLOS ONE

Additional Editor Comments (optional):

Reviewers' comments:

Reviewer's Responses to Questions

**Comments to the Author**

1. If the authors have adequately addressed your comments raised in a previous round of review and you feel that this manuscript is now acceptable for publication, you may indicate that here to bypass the “Comments to the Author” section, enter your conflict of interest statement in the “Confidential to Editor” section, and submit your "Accept" recommendation.

Reviewer #1: All comments have been addressed

Reviewer #3: All comments have been addressed

**********

2. Is the manuscript technically sound, and do the data support the conclusions?

The manuscript must describe a technically sound piece of scientific research with data that supports the conclusions. Experiments must have been conducted rigorously, with appropriate controls, replication, and sample sizes. The conclusions must be drawn appropriately based on the data presented. 

Reviewer #1: Yes

Reviewer #3: Yes

**********

3. Has the statistical analysis been performed appropriately and rigorously? 

Reviewer #1: Yes

Reviewer #3: Yes

**********

4. Have the authors made all data underlying the findings in their manuscript fully available?

The PLOS Data policy requires authors to make all data underlying the findings described in their manuscript fully available without restriction, with rare exception (please refer to the Data Availability Statement in the manuscript PDF file). The data should be provided as part of the manuscript or its supporting information, or deposited to a public repository. For example, in addition to summary statistics, the data points behind means, medians and variance measures should be available. If there are restrictions on publicly sharing data—e.g. participant privacy or use of data from a third party—those must be specified.

Reviewer #1: Yes

Reviewer #3: Yes

**********

5. Is the manuscript presented in an intelligible fashion and written in standard English?

PLOS ONE does not copyedit accepted manuscripts, so the language in submitted articles must be clear, correct, and unambiguous. Any typographical or grammatical errors should be corrected at revision, so please note any specific errors here.

Reviewer #1: Yes

Reviewer #3: Yes

**********

6. Review Comments to the Author

Please use the space provided to explain your answers to the questions above. You may also include additional comments for the author, including concerns about dual publication, research ethics, or publication ethics. (Please upload your review as an attachment if it exceeds 20,000 characters)

Reviewer #1: (No Response)

Reviewer #3: The authors have adequately addressed my comments.I feel that this manuscript is now acceptable for publication.

**********

7. PLOS authors have the option to publish the peer review history of their article (what does this mean?). If published, this will include your full peer review and any attached files.

If you choose “no”, your identity will remain anonymous but your review may still be made public.

**Do you want your identity to be public for this peer review?** For information about this choice, including consent withdrawal, please see our Privacy Policy.

Reviewer #1: No

Reviewer #3: No

# Editor report: Acceptance letter

**DOI:** 10.1371/journal.pone.0239592.r004

**Authors:** Kenji Hashimoto

17 Sep 2020

PONE-D-20-11603R1

Short-term effect of a smartphone application on the mental health of university students: A pilot study using a user-centered design self-monitoring application for mental health

Dear Dr. Kajitani:

I'm pleased to inform you that your manuscript has been deemed suitable for publication in PLOS ONE. Congratulations! Your manuscript is now with our production department.

If your institution or institutions have a press office, please let them know about your upcoming paper now to help maximize its impact. If they'll be preparing press materials, please inform our press team within the next 48 hours. Your manuscript will remain under strict press embargo until 2 pm Eastern Time on the date of publication. For more information please contact onepress@plos.org.

If we can help with anything else, please email us at plosone@plos.org.

Thank you for submitting your work to PLOS ONE and supporting open access.

Kind regards,

PLOS ONE Editorial Office Staff

on behalf of

Prof. Kenji Hashimoto 

Section Editor

PLOS ONE
